# Micropropagation and elicited production of triterpenoid saponin glycosides and stigmasterol *via* precursor and elicitor feeding in *Bacopa floribunda* (R.Br.) Wettst.—A potential nootropic herb

**DOI:** 10.3389/fpls.2023.1096842

**Published:** 2023-01-31

**Authors:** Shreedhar S. Otari, Suraj P. Devkar, Suraj B. Patel, Savaliram G. Ghane

**Affiliations:** Plant Physiology Laboratory, Department of Botany, Shivaji University, Kolhapur, Maharashtra, India

**Keywords:** plant growth regulators (PGRs), *Bacopa*, triterpenoid saponin glycosides, HPLC, nootropic, elicitation, methyl jasmonate (MeJA)

## Abstract

*Bacopa floribunda* (Family: Plantaginaceae) is used in folklore medicines for the management of cognitive dysfunction. It has nootropic, antiaging, anti-inflammatory, anti-cholinesterase, and antioxidant properties. We developed an efficient and reproducible protocol for *in vitro* propagation of *B. floribunda* using the nodal explants. We assessed the effects of Murashige and Skoog (MS) medium fortified with various plant growth regulatory substances (PGRs), a precursor, and elicitors and their optimal combinations on regeneration and production of total saponins, triterpenoid saponin glycosides (bacoside A3, bacopaside X, bacopaside II, and bacosaponin C), and stigmasterol content in *in vitro* grown biomass of *B. floribunda*. The growth of the shoots and roots was stimulated by MS + 2.0 mg/l BAP + 2.0 mg/l KIN and MS + 0.5 mg/l IAA + 0.5 mg/l IBA + 1.0 mg/l NAA, respectively. After 10 weeks of acclimatization, plantlets of *B. floribunda* had a survival rate of 95%. The highest total saponin content (35.95 ± 0.022 mg DE/g DW) was noted in the treatment of MS + 2.0 mg/l BAP + 1.5 μM SQ. Similarly, total triterpenoid saponin glycosides and stigmasterol were found maximum in biomass derived from MS + 2.0 mg/l BAP + 1.5 μM SQ and MS + 2.0 mg/l BAP, respectively. At the same treatments, bacoside A3 (1.01 ± 0.195 mg/g DW), bacopaside II (43.62 ± 0.657 mg/g DW), bacopaside X (1.23 ± 0.570 mg/g DW), bacosaponin C (0.19 ± 0.195 mg/g DW), and stigmasterol (7.69 ± 0.102 mg/g DW) were reported. The present findings will help to highlight *B. floribunda* as a potent memory-enhancing herb, and in future also, it could be a potential substitute to *B. monnieri*. The current work is the first to describe the micropropagation and the elicited production of bioactive metabolites from the *in vitro* grown biomass of *B. floribunda*. In addition, further research is needed on production of bioactives, their pharmacological effects, and the elicited production using callus, cell suspension, and hairy root cultures.

## Introduction

*Bacopa floribunda* (R.Br.) Wettest. (Family: Plantaginaceae) is indigenous to Madagascar, Africa, and Asia. In Ayurveda, it has been utilized as a brain tonic and antiaging and also to treat psychological disorders. It is an excellent source of natural medicine due to its cholinergic enzyme-inhibiting, anti-inflammatory, and antioxidant properties. Because of its potent nootropic effects, which decrease acetylcholinesterase (AChE) inhibition, it is used in traditional remedies to improve memory (Olatunji et al., 2019; Adetuyi et al., 2022). Triterpenoid saponin glycosides are said to be the active chemical components in the genus *Bacopa* and categorized as pseudojujubogenin (bacoside II and bacosaponin C) and jujubogenin glycosides (bacoside A3 and bacopaside X) and to exhibit memory-improving effects (Mathur et al., 2016; Nuengchamnong et al., 2016; Watcharatanon et al., 2019; Bhandari et al., 2020). Triterpenoid saponin glycosides from *B. monnieri* (Brahmi) are the well-known marker compounds used to improve nerve impulse transmission. Triterpenoid saponin glycosides help injured neurons’ recovery by promoting kinase activity, neuronal synthesis, and the restoration of synaptic function. It ultimately results in restoration of nerve impulse transmission and an increase in *de novo* synthesis of proteins in the brain. Nerve impulse transmission plays a crucial role in promoting healthy cognitive functions such as attention span, focus, concentration, learning, and memory (Mathur et al., 2016; Singh and Dhawan, 1997).

Due to its usage in the production of pharmaceuticals and herbal extracts, *B. monnieri* (Brahmi) is in very high demand in India, which promotes massive collection from their natural habitats and the extinction of the species. The greatest obstacle to the use of Brahmi in several industrial applications is the high variability in the levels of bioactive principles (Christopher et al., 2017). Since ancient times, *B. monnieri* (Brahmi) has been used as a brain tonic or memory booster. *Bacopa* formulations have been employed for therapeutic purposes by numerous academics and pharmaceutical firms worldwide, including those in India, New Zealand, the United States, and Australia. It has been estimated that approximately 10,000 t of fresh plant material was harvested from natural habitats each year that caused a significant decline in wild populations. Due to the presence of bioactive compounds, Brahmi has unique medicinal utility. Low yields, geographical and environmental variability, and other factors are linked to the synthesis and accumulation of secondary metabolites in the field-grown plants of Brahmi (Christopher et al., 2017; Watcharatanon et al., 2019; Asadollahei et al., 2022). Considering the problems associated with the growth and importance of the species, it is necessary to find out alternative sources and their mass multiplication, conservation, and production of bioactive metabolites. *In vitro* propagation makes it possible for cells, tissues, and organs to grow aseptically without the presence of microbes, which is crucial for the development of therapeutic plants (Ali et al., 2021; Bhattacharyya et al., 2022). The demand of providing high-quality-stock plants for conservation and pharmaceutical needs through micropropagation has remarkably increased (Komakech et al., 2020). *In vitro* produced cells, tissues, and organ cultures offered a good source of homogenous, sterile, and suitable materials for the conservation of plant species, their biochemical characterization, and the identification of bioactive constituents (Patil et al., 2013). *In vitro* propagation techniques have been used earlier to conserve several commercially important, ornamental, medicinal, and RET species (Nikam et al., 2009; Gupta et al., 2016; Chandra et al., 2020).

Recent studies have shown numerous methods for large-scale production; the synthesis of secondary metabolites includes strain improvement, optimization, elicitations, medium and culture environment manipulation, and nutrient and precursor feeding (Murthy et al., 2014). Precursors and elicitors can trigger morphological and physiological alterations that result in the accumulation of phytoalexins. Elicitation with abiotic and biotic treatments can also boost the production of bioactive metabolites (Watcharatanon et al., 2019). Elicitors have the ability to activate a cell immune system and produce secondary metabolites as a protection mechanism (Asadollahei et al., 2022). It is believed that adding precursors into the plant biosynthesis pathway will facilitate the production of secondary metabolites (Watcharatanon et al., 2019). The use of elicitors like methyl jasmonate (MJ), salicylic acid (SA), and precursors like squalene (SQ) for production of secondary metabolites in *in vitro* culture systems has been reported earlier (Largia et al., 2015; Baek et al., 2019; ). Several reports are available on micropropagation and enhanced metabolite production in *B. monnieri* (Banerjee and Shrivastava, 2008; Parale and Nikam, 2009; Parale et al., 2010; Largia et al., 2015; Sharma et al., 2015; Muzynska et al., 2016; Watcharatanon et al., 2019).

Although *B. floribunda* is a versatile medicinal herb, there are no reports available on its *in vitro* propagation and elicited production of bioactive compounds. Taking all of this into consideration, the objectives of the current study were to develop a micropropagation protocol and production of secondary metabolites. To achieve these goals, MS media supplemented with different plant growth regulatory substances (PGRs), precursors, and elicitors and their optimal concentrations were studied for the production of total saponins, four triterpenoid saponin glycosides, and stigmasterol.

## Materials and methods

### Standards and reagents

Standard triterpenoid saponin glycosides (bacoside A3, bacoside II, bacopaside X, and bacosaponin C) and stigmasterol of HPLC grade were purchased from Sigma-Aldrich and TCI Chemicals. Methanol, water, and acetonitrile (ACN) used in the analysis were of HPLC grade. All the marker compounds used in the study had a purity of above 95%. All the chemicals, nutrient media, gelling agents, PGRs, precursors, and elicitors were procured from Sigma-Aldrich (USA), TCI Chemicals (Tokyo, Japan), Sisco Research Laboratory (SRL), and HiMedia (Mumbai, India).

### Collection of plant materials and establishment of cultures

Plants of *Bacopa floribunda* (R.Br.) Wettest. were collected from the rice fields of Rajapur, Maharashtra, India, during March 2021. The sample specimen was deposited in an herbarium (SUK) of the Department of Botany, Shivaji University, Kolhapur, Maharashtra (Voucher no. SSO 032). The plants were brought to the laboratory, carefully washed under running water, then treated with 0.1% (v/v) Tween-20 for 5 min and rinsed five times with sterile distilled water. These explants (nodal segments) were further surface sterilized by first rinsing them with aqueous HgCl_2_ (0.1% w/v) for 4 min and then washed five times with sterile distilled water to remove the traces of HgCl_2_. Initially, cultures were established on MS medium (1962) devoid of any PGRs (Murashige and Skoog, 1962). In further investigations, different PGRs including auxins (IAA, IBA, and NAA), cytokinins (BAP, and KIN), a precursor (squalene, SQ), and elicitors [methyl jasmonate (MJ), salicylic acid (SA), potassium chloride (KCl), and magnesium sulfate (MgSO_4_)] were added to MS medium. All the surface-sterilized nodal explants (1.0 cm) were excised and then transferred to a semisolid nutrient medium under aseptic conditions. All nutrient media used in the study contained vitamins and also supplemented with 3% sucrose, with a pH level maintained at 5.8 ± 0.5, solidified with 8 g/l agar, and autoclaved at 121°C for 15 min. All cultures in this experiment were maintained under controlled conditions (25 ± 2°C temperature, 80 ± 10% relative humidity, 8-h photoperiod, and 30–40 μmol m^-2^ S^-1^ light intensity).

### Effects of different PGRs on shoot and root regeneration

Initially, cultures were established on MS medium devoid of any PGRs. Further, healthy nodal explants (1.0 cm) were carefully excised and inoculated on MS medium (50 ml) fortified with different concentrations (0.5, 1.0, 1.5, and 2.0 mg/l) of cytokinins (BAP, KIN). Likewise, varied levels of auxins (IAA, IBA, and NAA; 0.5, 1.0, 1.5, and 2.0 mg/l) were used.

### Rooting of shoots and acclimatization of plantlets

MS medium enriched with the optimal concentrations of auxins was used, and rooting of shoots was studied after 3 weeks of culture. Shoots with multiple roots from the abovesaid medium were acclimatized under field conditions. Well-grown rooted shoots were carefully removed from the culture bottles, and surplus culture media were removed carefully. They were also delicately moved into the plastic pots containing soil and coco pit mixture (1:1). To maintain humidity (80–90%), all the plantlets were covered with transparent polythene bags. These pots were kept inside a greenhouse for 2 weeks. Controlled conditions were maintained as 50 μmol m^-2^ S^-1^ light intensity, 25 ± 5°C temperature, and 80 ± 10% relative humidity. Initially, half-strength MS was given to the plantlets for a period of 2 weeks and then watered regularly. After 3 weeks, polythene bags were removed and plantlets were exposed to natural conditions. After 10 weeks, the survival rate was calculated and expressed as percentage.

### Effects of PGRs, precursors, and elicitors on bioactive metabolites (total triterpenoid saponin glycosides and stigmasterol)

In the study, different elicitors, *viz*., methyl jasmonate (MJ; 30, 60, 80, 100 μM), salicylic acid (SA; 30, 60, 80, 100 μM), potassium chloride (KCl; 30, 60, 80, 100 mM), and magnesium sulfate (MgSO_4_; 30, 60, 80, 100 mM) were added separately to the nutrient medium. Likewise, squalene alone (SQ; 0.5, 1.0, 1.5, 2.0 μM) was used as a precursor. Further, the influence of optimal concentrations of auxins and cytokinins in combination with the optimal concentration of the precursor was used and the contents of triterpenoid saponin glycosides and stigmasterol were studied. Details of the treatments used were as follows: MS + 2.0 mg/l BAP + 2.0 mg/l KIN (OCC^*^), MS + 0.5 mg/IAA + 0.5 mg/IBA + 1.0 mg/l NAA (OCA^*^), MS + 2.0 mg/BAP + 2.0 mg/l KIN + 1.5 μM SQ (OCC^*^P^°^), MS + 0.5 mg/l IAA + 0.5 mg/l IBA + 1.0 mg/l NAA + 1.5 μM SQ (OCA^*^P^°^), MS + 2.0 BAP mg/l + 1.5 μM SQ (OCC^*^P1^°^), MS + 2.0 mg/l KIN + 1.5 μM SQ (OCC^*^P2^°^),MS + 1.0 mg/l NAA + 2.0 mg/l KIN + 1.5 μM SQ (OCAC^*^P^°^), and MS + 1.0 mg/l NAA + 2.0 mg/l KIN (OCAC^*^).

In the present study, nodal explants (four explants per culture bottle and 10 bottles for each treatment) initially grown on plane MS medium were used. All cultures in this experiment were maintained as per the abovementioned conditions. After a culture period of 21 days, several parameters such as average shoot length, number of leaves per shoot, fresh and dry weights, number of roots, average root length, and percent regeneration were determined.

### Analysis of total saponins, triterpenoid saponin glycosides, and stigmasterol contents

#### Preparation of samples and standard solutions

An ultrasonicator (Rivotek, India) was used to extract the saponins and stigmasterol. *In vitro* grown and hardened plant biomass (0.5 g) were mixed with 10 ml of methanol and subjected to ultrasonic assisted extraction for a period of 10 min. The homogenate was then centrifuged at 10,000 rpm (Emtek Instruments, India). Further, the supernatant was collected and condensed and the volume adjusted to 1 ml using the same solvent. Standard compounds including diosgenin, triterpenoid saponin glycosides, and stigmasterol were carefully weighed (1 mg) and dissolved separately in HPLC-grade methanol (1 ml) to obtain a standard stock solution (mg/ml) (Careri et al., 2001; Bhandari et al., 2006). The preparation of working and stock solutions included dilution of the stock solution with the appropriate solvent to provide six distinct concentrations. All of the extracts and working and stock solutions were stored at 4°C until further use. Prior to HPLC analysis, all the standards and extracts were filtered using 0.22-μm nylon filters (HiMedia, India). A standard curve with different concentrations (20–100 µg/ml) was prepared, and results were expressed as mg/g DW.

#### Estimation of total saponin content

Total saponin content (TSC) was evaluated by using the protocol of Uysal et al. (2017) with slight modifications. A plant extract (200 µl) in mg/ml was mixed with 200 µl of 8% vanillin, and 1,000 µl of 72% H_2_SO_4_ acid was added to it; the reaction mixture was further incubated for 10 min in a water bath at 60°C (Equitron, India). After complete incubation, reaction mixtures were cooled for 15 min and absorbance was recorded at 544 nm using a UV-vis spectrophotometer (Jasco V-730, Japan). Diosgenin was used as a standard, and results were represented as mg diosgenin equivalent (DE)/g DW.

#### Estimation of triterpenoid saponin glycosides using HPLC

The Jasco LC-2000 Plus chromatographic system, which has a binary pump, an autosampler, and a UV detector (UV-2070), was used for the detection of triterpenoid saponin glycosides. Utilizing a Hiber C18 column (5 μM, 250 X 4.6 mm), separation of bacoside A3, bacoside II, bacopaside X, and bacosaponin C was carried out. Built-in ChromNAV software was utilized for data processing. A flow rate of 0.8 ml/min, an injection volume of 20 μl, and a mobile phase consisting of water:acetonitrile (70:30 v/v) was used (Bhandari et al., 2006). The peaks were observed at 205 nm with a run time of 20 min. The amount of respective saponins was calculated by comparing the chromatogram with standard and expressed as milligrams per gram of dry weight (mg/g DW).

### HPLC analysis of stigmasterol

Detection of stigmasterol was carried out using the same instrumentation system as specified above. An isocratic mobile phase consisting acetonitrile:water (70:30 v/v) was used with a 1-ml/min flow rate, a 20-μl injection volume, and a 20-min run period. The peaks from standard and samples were monitored at 210 nm (Careri et al., 2001). By comparing their retention time with those of the standard, stigmasterol from the extracts was identified. The amount of stigmasterol was calculated and expressed as milligrams per gram of dry weight (mg/g DW).

By spiking with known concentrations of the respective standards, triterpenoid saponin glycosides and stigmasterol were verified. Samples and standards were analyzed in triplicates to improve the quality of the results.

### Statistical analysis

All the analyses were performed in triplicate, and values were represented as mean ± standard error. The data were subjected to one-way analysis of variance (ANOVA), and significant differences between mean values were determined by Duncan’s multiple-range test (P < 0.05) using SPSS software ver. 16. Principal component analysis (PCA) was used to analyze the data produced from the investigated morphological responses and bioactive compounds from various concentrations of PGRs, precursors, and elicitors using Minitab software (trial ver. 19).

## Results

### *In vitro* micropropagation of *B. floribunda*

**Figure 1:**
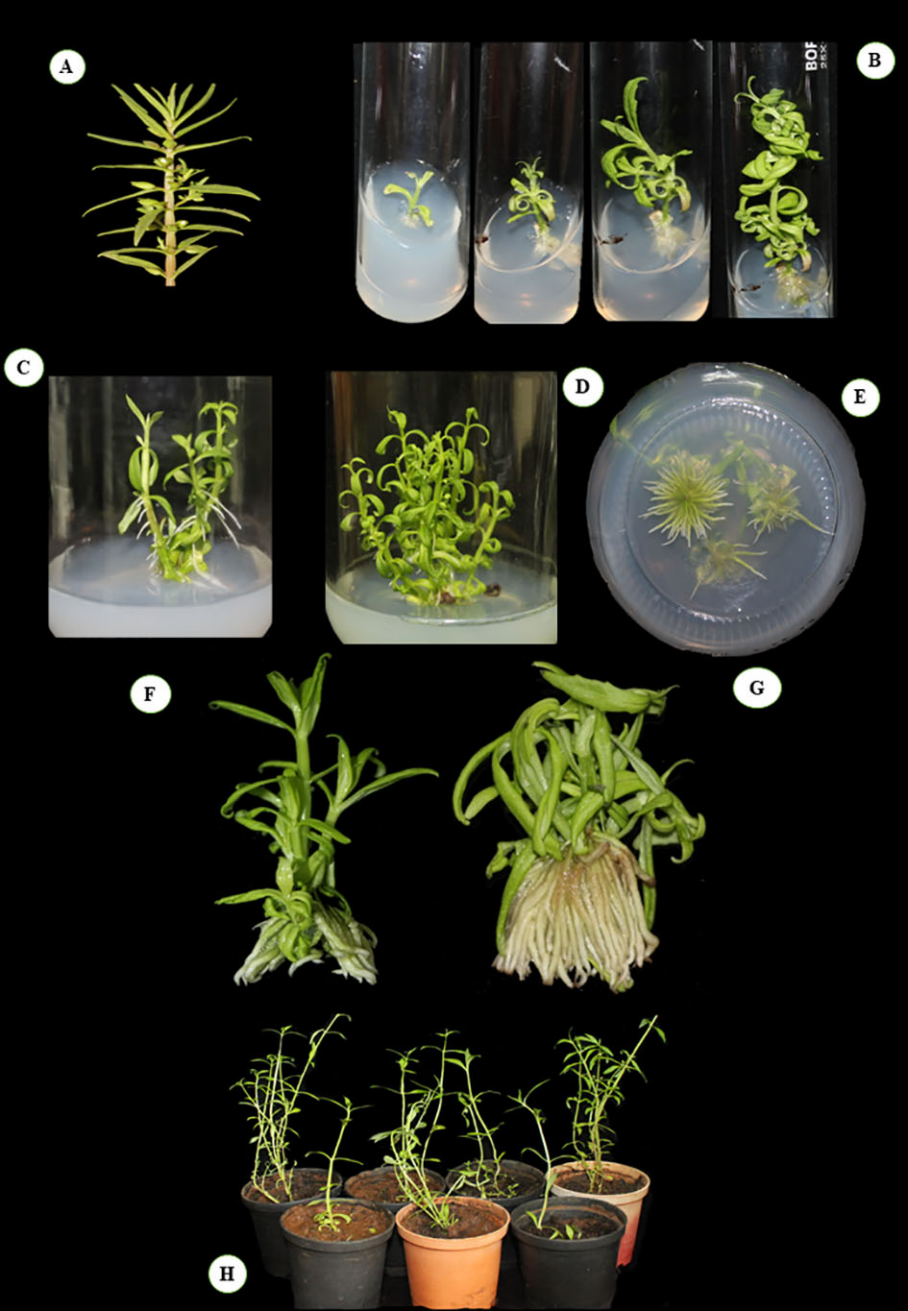
Different stages of micropropagation in *Bacopa floribunda*. **(A)** Mother plant; **(B)** various stages of caulogenesis. **(C, D)** Development of multiple shoots on cytokinin containing media. **(E)** Rooting of shoots. **(F)** Initiations of roots in optimum combination auxins. **(G)** Formation of multiple roots using optimum auxins. **(H)** Hardened plants (10 weeks old).

In the study, the influence of different cytokinins (BAP and KIN) on various parameters was tested and results are depicted in **Table 1** and **Figure 1**. A reliable protocol for direct *in vitro* propagation using fresh nodal segments of *B. floribunda* was standardized (**Figure 1A**). After a period of 21 days, cytokinins (BAP, KIN) at all concentrations (0.5, 1.0, 1.5, and 2.0 mg/l) denoted the improved shoot growth. Nodal explants of *B. floribunda* cultured on the higher concentrations of BAP and KIN (2.0 mg/l) revealed 100% regeneration. Among all the different PGRs tested, the highest number of leaves per shoot (15.60 ± 0.40) with a maximum shoot length (6.4 ± 0.40 cm) was obtained in MS medium supplemented with 2.0 mg/l KIN (**Table 1**, **Figure 1B** **)**. However, the shoot growth depended on the type and concentration of cytokinin. The statistical analysis revealed that among all the cytokinin concentrations, MS + 2.0 mg/l KIN resulted in a considerably higher shoot length (6.4 ± 0.40 cm). MS + 2.0 mg/l BAP also demonstrated the second highest enhancement in shoot length (5.4 ± 0.40 cm). However, nodal explants treated with MS + 1.0 mg/l BAP had the shortest shoot length (3.95 ± 0.22 cm). Similarly, MS + 2.0 mg/l KIN revealed the highest number of leaves per shoot (15.60 ± 0.40), followed by MS + 2.0 mg/l BAP with 15.2 ± 0.49 leaves per shoot. In contrast, MS + 1.0 mg/l BAP exhibited the lowest number of leaves per shoot (9.0 ± 0.58). The maximum fresh (2.93 ± 0.5 g) and dry (0.150 ± 0.02 g) weights were found in the cultures grown on MS + 2.0 mg/l BAP (**Table 1**). The highest concentrations of both the cytokinins revealed superior responses in terms of the axillary shoot initiation, number of leaves, and fresh and dry weights in the cultures of *B. floribunda*. These cultures were further maintained on the medium supplemented with cytokinins for a period of 2 months by repeated subculturing, and multiple shoots with the roots were observed (**Figure 1C**). These well-developed shoots maintained on the same medium resulted into multiple-shoot formation (**Figure 1D**). Shoots grown on basal MS medium were further subjected to different concentrations of IAA, IBA, and NAA (0.5–2.0 mg/l), and results are presented in **Table 2** and **Figure 1E**. The tested concentrations of auxins promoted the root growth after 21 days of incubation. The root initiation and growth depend upon the auxin type and its concentration. Results showed that among the auxins used at different concentrations, MS + 1.0 mg/l NAA produced significantly higher root numbers (32 ± 1.35) and also showed the highest average root length (2.6 ± 0.16 cm). In contrast, levels of NAA (0.5 and 2.0 mg/l) showed the lowest number of roots per shoot (4.0 ± 0.10) with an average root length of 1.9 ± 0.11 cm. The roots that were initiated using MS + 1.0 mg/l NAA were more robust and tightly connected to the base of the well-grown shoots. Levels of other auxins (0.5 and 1.5 mg/l IAA, 0.5 and 2.0 mg/l IBA, and 1.0 and 1.5 mg/l NAA) failed to affect the root length in a significant way. Incorporation of auxins in the nutrient medium significantly affected the fresh weight (FW) that ranged between 1.3 and 2.75 g wherein MS + 1.0 mg/l NAA indicated the maximum fresh weight. Similarly, maximum dry weight (DW) was reported when the MS medium was enriched with 0.5 mg/l IAA (0.130 ± 0.02 g), followed by 1.0 mg/l NAA and 0.5 mg/l IBA. Our results highlighted that MS fortified with 1.0 mg/l NAA showed the best initiation and growth of roots (**Table 2**, **Figure 1E**). In the case of cytokinins, response of KIN was comparatively higher than that of BAP. Similarly, among all the auxins used, NAA was found to be the most responsive followed by IAA and IBA (**Table 2**).

**Table 1:** Effect of cytokinins (BAP and KIN) on regeneration of *Bacopa floribunda*.

Cytokinins (mg/l)		Average shoot length (cm)	No. of leaves per shoot	No. of roots per shoot	Average root length (cm)	FW (g)	DW (g)	Response (%)
**BAP**	0.5	5.13 ± 0.97^abc^	11.33 ± 0.67^bc^	15.67 ± 4.70^a^	3.23 ± 0.33^a^	1.74 ± 0.04^ab^	0.275 ± 0.07^a^	90^c^
	1.0	3.95 ± 0.22^c^	9.00 ± 0.58^c^	10.00 ± 0.58^b^	2.90 ± 0.20^a^	0.75 ± 0.03^b^	0.108 ± 0.01^b^	95^b^
	1.5	4.20 ± 0.20^c^	10.40 ± 0.40^bc^	6.40 ± 0.40^c^	3.20 ± 0.20^a^	2.44 ± 0.43^a^	0.114 ± 0.04^b^	100^b^
	2.0	5.40 ± 0.40^ab^	15.20 ± 0.49^a^	9.20 ± 0.49^bc^	3.40 ± 0.24^a^	2.93 ± 0.50^a^	0.150 ± 0.02^b^	100^a^
**KIN**	0.5	5.12 ± 0.71^abc^	12.00 ± 1.15^b^	13.20 ± 0.75^ab^	3.40 ± 0.28^a^	1.52 ± 0.40^ab^	0.109 ± 0.01^b^	95^b^
	1.0	5.38 ± 0.21^abc^	12.40 ± 0.81^ab^	13.40 ± 0.87^ab^	3.50 ± 0.26^a^	1.35 ± 0.30^ab^	0.114 ± 0.01^b^	100^a^
	1.5	4.60 ± 0.24^abc^	12.80 ± 0.49^ab^	13.20 ± 0.49^ab^	3.40 ± 0.24^a^	1.90 ± 0.10^ab^	0.140 ± 0.02^b^	100^a^
	2.0	6.40 ± 0.40^a^	15.60 ± 0.40^a^	14.40 ± 1.17^a^	3.60 ± 0.24^a^	2.20 ± 0.32^ab^	0.133 ± 0.01^b^	100^a^

Values represent the averages and standard errors of three independent measurements. According to Duncan’s multiple-range test, mean values in the same column with various alphabets exhibited statistically significant differences.

**Table 2:** Effect of auxins (IAA, IBA, and NAA) on regeneration of *Bacopa floribunda*.

Auxins (mg/l)		Average shoot length (cm)	No. of leaves per shoot	No. of roots per shoot	Average root length (cm)	FW (g)	DW (g)	Response (%)
**IAA**	0.5	5.1 ± 071^bc^	10 ± 0.63^ab^	19.4 ± 2.25^a^	2.6 ± 0.15^a^	2.28 ± 0.1^ab^	0.130 ± 0.002^a^	100^a^
	1.0	4.3 ± 0.17^bc^	8.4 ± 0.40^ab^	18.4 ± 0.87^bc^	2.2 ± 0.07 ^ab^	1.46 ± 0.30^ab^	0.114 ± 0.005^a^	100^a^
	1.5	3.0 ± 0.10^c^	8.4 ± 0.40^ab^	11.8 ± 0.4^d^	2.6 ± 0.20^a^	1.80 ± 0.17^ab^	0.121 ± 0.006^a^	100^a^
	2.0	3.0 ± 0.10^c^	8.5 ± 0.5^ab^	11.75 ± 0.5^d^	2.25 ± 0.3 ^ab^	1.71 ± 0.2^ab^	0.126 ± 0.005^a^	100^a^
**IBA**	0.5	6.9 ± 0.71^a^	12.8 ± 0.49^a^	18.6 ± 1.25^bc^	2.6 ± 0.18^a^	1.80 ± 0.2^ab^	0.122 ± 0.007^a^	100^a^
	1.0	6.8 ± 0.53^a^	10.4 ± 0.75^ab^	13.4 ± 0.75^b^	2.1 ± 0.11^ab^	1.70 ± 0.11^ab^	0.116 ± 0.001^a^	100^a^
	1.5	3.0 ± 0.10^c^	12.6 ± 0.40^a^	16.6 ± 0.67^c^	2.4 ± 0.058 ^ab^	1.30 ± 0.28^b^	0.118 ± 0.001^a^	90^b^
	2.0	3.0 ± 0.10^c^	12.4 ± 0.49^a^	13.2 ± 0.49^d^	2.6 ± 0.11 ^a^	1.76 ± 0.02^ab^	0.118 ± 0.001^a^	100^a^
**NAA**	0.5	5.5 ± 0.76^b^	11.0 ± 0.52^a^	20.0 ± 2.03^b^	1.9 ± 0.11 ^ab^	1.42 ± 024^ab^	0.117 ± 0.001^a^	90^b^
	1.0	6.8 ± 0.72^a^	9.5 ± 2.50^ab^	32.0 ± 1.35^a^	2.6 ± 0.16 ^a^	2.75 ± 0.45^a^	0.124 ± 0.002^a^	100^a^
	1.5	3.0 ± 0.10^c^	7.2 ± 0.49^b^	4.0 ± 0.10^c^	2.6 ± 0.24 ^a^	2.04 ± 0.20^ab^	0.113 ± 0.001^a^	100^a^
	2.0	3.0 ± 0.10^c^	7.6 ± 0.89^b^	4.0 ± 0.10^c^	2.2 ± 0.45^ab^	1.62 ± 0.20^ab^	0.107 ± 0.001^a^	100^a^

Values represent the averages and standard errors of three independent measurements. According to Duncan’s multiple-range test, mean values in the same column with various alphabets exhibited statistically significant differences.

### Regeneration responses of *B. floribunda* using precursors and elicitors

The precursor (squalene) and elicitors (methyl jasmonate, salicylic acid, potassium chloride, magnesium sulfate) were added to MS, and growth responses were studied (**Table 3**). Incorporation of the precursor and elicitors showed the variations in the studied responses (average shoot length, number of leaves per shoot, number of roots per shoot, average root length, FW, DW, and percent response). Our results highlighted that within the studied levels of precursor and elicitors, the MS medium enriched with 1.5 μM SQ, 30 μM SA, 80 μM MJ, 30 mM MgSO_4_, and 60 mM KCl showed the highest responses. The average shoot length of 3 to 5 cm was reported at all the concentrations of precursor and elicitors used. The highest shoot length (5.2 ± 0.22 cm) and maximum number of leaves (18 ± 0.71 cm) were recorded in MS + 1.5 μM SQ, whereas addition of 30 mM KCl to the medium revealed the lowest responses (3.0 ± 0.24 cm and 8.4 ± 0.40, respectively). In contrast, the highest number of roots (18.0 ± 0.75) with maximum root length (3 ± 0.10 cm) was observed from the squalene-enriched medium (MS + 1.5 μM SQ). In the study, MS + 100 mM KCl denoted the minimum root number (4.6 ± 1.0) and root length (2.0 ± 0.10 cm). Incorporation of MJ, SA, and MgSO_4_ in the nutrient medium showed intermediate responses (**Table 3**). Moreover, the highest FW and DW were noted from the biomass raised on MS medium amended with 1.5 μM SQ. After analyzing the current findings, it was noted that squalene at a concentration of 1.5 μM promoted the *in vitro* growth of *B. floribunda* (**Table 3** **).**

**Table 3:** Effect of precursor (squalene) and elicitors (methyl jasmonate, salicylic acid, potassium chloride, and magnesium sulfate) on regeneration of *Bacopa floribunda*.

Precursors and elicitors		Average shoot length (cm)	No. of leaves per shoot	No. of roots per shoot	Average root length (cm)	FW (g)	DW (g)	Response (%)
**SQ** **(μM)**	0.5	5.0 ± 0.12^ab^	15.3 ± 0.67^bc^	15.3 ± 0.64^abc^	2.6 ± 0.33^b^	1.40 ± 0.12^bcd^	0.120 ± 0.01^a^	90^c^
	1.0	4.8 ± 0.49^abc^	17.6 ± 0.75^ab^	15.6 ± 0.71^ab^	2.6 ± 0.24^b^	1.35 ± 0.31^bcd^	0.119 ± 0.02^a^	100^a^
	1.5	5.2 ± 0.22^a^	18.0 ± 0.70^a^	18.0 ± 0.75^a^	3.0 ± 0.10^a^	2.10 ± 0.06^a^	0.126 ± 0.01^a^	100^a^
	2.0	3.0 ± 0.10^e^	9.6 ± 0.40^fghi^	8.0 ± 0.10^efg^	2.4 ± 0.24^b^	1.45 ± 0.10^bcd^	0.120 ± 0.04^a^	100^a^
**MJ** **(μM)**	30	3.0 ± 1.3^e^	12.4 ± 5.50^cdef^	7.6 ± 3.4^fgh^	3.0 ± 1.30^b^	1.08 ± 0.12^cdefg^	0.110 ± 0.01^a^	100^a^
	60	3.8 ± 0.20^de^	13.6 ± 075^cdef^	11.6 ± 0.40^def^	3.2 ± 0.10^a^	1.16 ± 0.10^bcdef^	0.120 ± 0.01^a^	100^a^
	80	4.4 ± 0.24^abc^	17.6 ± 0.75^ab^	14.6 ± 0.75^abcd^	3.2 ± 1.3^a^	1.54 ± 0.11^b^	0.140 ± 0.03^a^	100^a^
	100	4.0 ± 0.10^cde^	14.0 ± 0.50^bcd^	11.6 ± 1.6^def^	3.0 ± 0.10^b^	0.768 ± 0.18^fghi^	0.108 ± 0.04^a^	100^a^
**SA** **(μM)**	30	5.0 ± 0.10^ab^	17.6 ± 1.17^ab^	13.2 ± 0.49^cde^	3.0 ± 0.10^b^	0.806 ± 0.37^efghi^	0.102 ± 0.02^a^	100^a^
	60	3.0 ± 0.10^e^	12 ± 0.63^cdefg^	8.8 ± 0.49^ef^	2.0 ± 0.10^b^	0.680 ± 0.033^ghi^	0.100 ± 0.02^a^	100^a^
	80	3.8 ± 0.10^de^	12.4 ± 0.40^cdefg^	8.8 ± 0.49^ef^	2.0 ± 0.10^b^	0.790 ± 0.07^fghi^	0.100 ± 0.03^a^	100^a^
	100	3.0 ± 0.10^e^	11.6 ± 0.40^defgh^	4.8 ± 0.49^i^	2.2 ± 0.10^b^	0.420 ± 0.00^i^	0.100 ± 0.02^a^	100^a^
**KCl (mM)**	30	3.0 ± 0.24^e^	8.4 ± 0.40^hi^	5.4 ± 0.49^hi^	2.0 ± 0.10^b^	0.940 ± 0.07^defgh^	0.100 ± 0.02^a^	100^a^
	60	3.4 ± 0.20^e^	12.8 ± 0.49^cdef^	10.8 ± 0.40^cde^	2.4 ± 0.24^b^	1.40 ± 0.01^bcd^	0.129 ± 0.01^a^	100^a^
	80	3.2 ± 0.20^e^	12.4 ± 0.40^cdef^	10.4 ± 0.24^cde^	2.0 ± 0.10^b^	0.720 ± 0.04^fghi^	0.100 ± 0.02^a^	100^a^
	100	3.2 ± 0.4^e^	10 ± 0.60^efghi^	4.6 ± 1.0^hi^	2.0 ± 0.10^b^	0.900 ± 0.01^dfghi^	0.100 ± 0.01^a^	100^a^
**MgSO_4_** **(mM)**	30	4.2 ± 0.4^cde^	16.0 ± 1.40^bc^	15 ± 1.0^ab^	3.0 ± 0.10^b^	1.360 ± 0.26^bcd^	0.136 ± 0.01^a^	100^b^
	60	4.0 ± 0.01^cde^	13.0 ± 0.58^cdef^	11.5 ± 0.5^cdef^	2.5 ± 0.29^b^	1.024 ± 0.12^bcde^	0.100 ± 0.06^a^	100^b^
	80	3.25 ± 0.30^e^	11.5 ± 0.50^defgh^	8.5 ± 0.5^ef^	3.0 ± 0.10^b^	1.00 ± 0.06^cdefgh^	0.110 ± 0.02^a^	90^a^
	100	3.0 ± 0.10^e^	10.5 ± 0.50^defgh^	7.5 ± 0.5^fgh^	2.75 ± 0.25^b^	0.775 ± 0.14^fghi^	0.120 ± 0.02^a^	80^a^

Values represent the averages and standard errors of three independent measurements. According to Duncan’s multiple-range test, mean values in the same column with various alphabets exhibited statistically significant differences.

### Combined effects of optimal cytokinins, auxins, and precursors on *in vitro* regeneration

Based on the findings from **Tables 1** **–** **3**, the optimal concentrations of auxins, cytokinins, and squalene were used in combinations, and results are presented in **Table 4**. It was revealed that combinations of cytokinins, auxins, and precursors together altered the regeneration responses in *B. floribunda*. According to the statistical study, MS + 2.0 mg/l BAP + 1.5 μM SQ produced longer shoots (5.12 ± 0.71 cm) than the rest of the combinations. Likewise, MS + 2.0 mg/l BAP + 2.0 mg/l KIN exhibited the highest leaves per shoot (22.33 ± 1.45), followed by MS + 2.0 mg/l BAP + 2.0 mg/l KIN + 1.5 μM SQ, which had the second highest number of leaves (21.6 ± 1.17). However, the type and concentration of auxin determined the initiation and further elongation of roots. Findings revealed that MS + 0.5 mg/l IAA + 0.5 mg/l IBA + 1.0 mg/l NAA produced considerably maximum number of roots (36.0 ± 0.89) with average root length (3.5 ± 0.04 cm). The roots that were grown in the same medium were stronger and closely affixed to the base of the plantlets. Referring to the above findings, it was found that the optimal combination of auxins (MS + 0.5 mg/l IAA + 0.5 mg/l IBA + 1.0 mg/l NAA) was found to be the most ideal for root initiation (**Figure 1G**). In comparison with other treatments, the optimal combination of cytokinins (MS + 2.0 mg/l BAP + 2.0 mg/l KIN) had the highest FW (4.33 ± 0.75 g) and DW (0.191 ± 0.08 g), followed by MS + 0.5 mg/l IAA + 0.5 mg/l IBA + 1.0 mg/l NAA (3.09 ± 0.44 and 0.114 ± 0.01 g, respectively). Development of shoots, number of leaves, FW, DW, maximum root number, root length, and root development was observed in the cultures when the optimum combinations of cytokinins (MS + 2.0 mg/l BAP + 2.0 mg/l KIN) and auxins (MS + 0.5 mg/l IAA + 0.5 mg/l IBA + 1.0 mg/l NAA) were used. From the above results, it was observed that growth parameters (number of roots per shoot and leaves per shoot) in *B. floribunda* were significantly enhanced by using optimum combinations of auxins and cytokinins (**Table 4**, **Figures 1F, G**).

**Table 4:** Effect of optimal concentrations and combinations of PGRs and precursor on regeneration of *Bacopa floribunda*.

PGRs and precursor (mg/l–μM)	Average shoot length (cm)	No. of leaves per shoot	No. of roots per shoot	Average root length (cm)	FW (g)	DW (g)	Response (%)
OCC^*^	2.33 ± 0.33^de^	22.33 ± 1.45^a^	3.67 ± 0.4^c^	1.0 ± 0.10^c^	4.33 ± 0.75^a^	0.191 ± 0.08^a^	100^a^
OCA^*^	3.00 ± 0.10^cd^	15.75 ± 0.58^b^	36 ± 0.89^a^	3.5 ± 0.04^a^	3.09 ± 0.44^ab^	0.114 ± 0.01^ab^	100^a^
OCC^*^P^°^	4.0 ± 0.10^ab^	21.6 ± 1.17^a^	10 ± 0.63^bc^	2.16 ± 0.10^b^	1.79 ± 0.13^b^c	0.105 ± 0.01^b^	80^b^
OCA^*^P^°^	1.9 ± 0.10^e^	11.2 ± 1.02^cd^	12.4 ± 0.75^b^	2.2 ± 0.20^b^	1.44 ± 0.4^bc^	0.112 ± 0.01^b^	100^a^
OCC^*^P1^°^	5.12 ± 0.71^a^	12.0 ± 1.15^bc^	4.8 ± 1.22^c^	2.2 ± 0.20^b^	2.88 ± 0.19^ab^	0.111 ± 0.01^b^	100^a^
OCC^*^P2^°^	3.2 ± 0.20^bc^	12.4 ± 0.81^bc^	13.4 ± 0.87^b^	2.64 ± 0.14^ab^	1.85 ± 0.45^bc^	0.113 ± 0.01^b^	80^b^
OCAC^*^P^°^	2 ± 0.10^e^	7.6 ± 0.49^e^	13.2 ± 0.49^b^	2.6 ± 0.24^ab^	0.470 ± 0.08^c^	0.048 ± 0.01^b^	100^a^
OCAC^*^	2.6 ± 0.40^de^	15.6 ± 0.40^bc^	4.2 ± 0.66^c^	2.52 ± 0.18^ab^	1.44 ± 0.01b^c^	0.077 ± 0.02^b^	100^a^

^*^(mg/l), **^°^** (μM)

OCC^*^ - MS + 2.0 mg/l BAP + 2.0 mg/l KIN, OCA^*^ - MS + 0.5 mg/IAA + 0.5 mg/IBA + 1.0 mg/l NAA, OCC^*^P^°^ - MS + 2.0 mg/BAP + 2.0 mg/l KIN + 1.5 μM SQ, OCA^*^P^°^ - MS + 0.5 mg/l IAA + 0.5 mg/l IBA + 1.0 mg/l NAA + 1.5 μM SQ, OCC^*^P1^°^- MS + 2.0 BAP mg/l + 1.5 μM SQ, OCC^*^P2^°^- MS + 2.0 mg/l KIN + 1.5 μM SQ, OCAC^*^P^°^ - MS + 1.0 mg/l NAA + 2.0 mg/l KIN + 1.5 μM SQ, OCAC^*^ - MS + 1.0 mg/l NAA + 2.0 mg/l KIN.

Values represent the averages and standard errors of three independent measurements. According to Duncan’s multiple-range test, mean values in the same column with various alphabets exhibited statistically significant differences.

### Acclimatization of plantlets

Nutrient medium amended with the optimal auxin combinations, i.e., MS + 0.5 mg/l IAA + 0.5 mg/l IBA + 1.0 mg/l NAA, showed the best rooting responses. After 3 weeks of cultures, *in vitro* grown rooted shoots were taken out from the culture vessels and used for acclimatization. After 10 weeks of acclimatization, *in vitro* regenerated plantlets of *B. floribunda* had a survival rate of 95%. The acclimatized plants grew well and were morphologically identical to the mother plants (**Figure 1H**). The total height of hardened plantlets (10 weeks old) was approximately 8 to 10 cm with 13–18 leaves per plant. The acclimatized *in vitro* plantlets showed no evident variation in morphology or growth characteristics (**Figure 1H**).

### Estimation of total saponins, triterpenoid saponin glycosides, and stigmasterol contents from *in vitro* grown biomass

**Figure 2:**
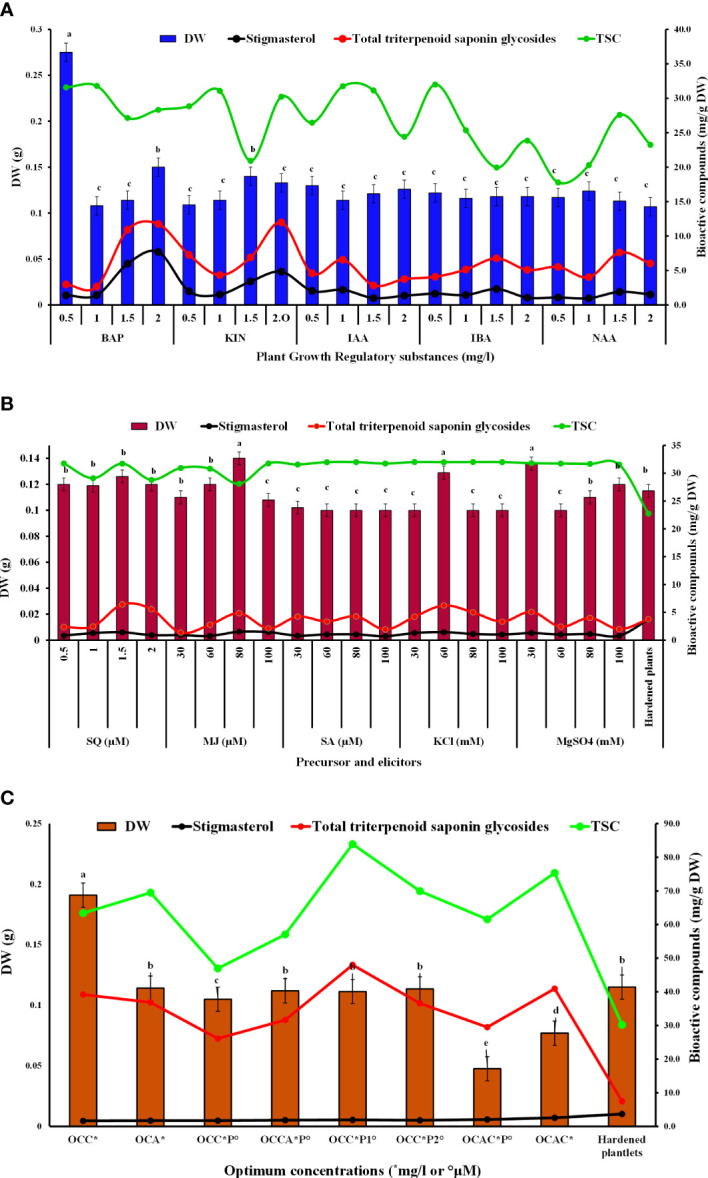
Effects of different PGRs **(A)**, precursor and elicitors **(B)**, and their optimal combinations **(C)** on dry weight, total saponins, total triterpenoid saponin glycosides, and stigmasterol contents from *in vitro* grown biomass of *B. floribunda*.

*In vitro* biomass derived from the experiments (auxins, cytokinins, precursor, and elicitors and their combinations) were subjected to estimation of various bioactive compounds, particularly total saponins (TSC), triterpenoid saponin glycosides, and stigmasterol using spectrophotometry and HPLC (**Figure 2**).

### Total saponin content

TSC from the *in vitro* grown biomass was determined using a spectrophotometer, and results are represented in **Figure 2**. Fortification of PGRs, precursor, and elicitors in MS showed reliable TSC which was found in the range of 17 to 32 mg/g DW. Among all the levels of cytokinins, MS + 1.5 mg/l KIN showed the least TSC (20.92 ± 4.8 mg DE/g DW), whereas MS + 1.0 mg/l BAP exhibited the highest content (31.79 ± 0.011 mg DE/g DW) followed by MS + 0.5 mg/l BAP and MS + 1.0 KIN mg/l (**Figure 2A**). In the case of auxins, MS + 0.5 mg/l IBA was found to be the best medium for total saponin content (32.0 ± 0.001 mg DE/g DW), whereas the lowest content (17.78 ± 2.1 mg DE/g DW) was found in NAA (0.5 mg/l) containing medium (**Figure 2A**). Additionally, when compared with other concentrations of the studied precursor and elicitors, MS + 1.5 μM SQ was found to be the best treatment that offered the highest TSC (31.7 ± 0.04 mg DE/g DW). Furthermore, in the elicitors tested, addition of KCl in the nutrient medium showed the highest TSC, followed by SA, MgSO_4_, and MJ (**Figure 2B**). MS modified with 80 mM KCl showed the highest TSC (32.01 ± 0.10 mg DE/g DW), whereas the least response (28.10 ± 0.079 mg DE/g DW) was observed when 80 mM MJ was added in the medium. Other treatments including SA, MgSO_4_, and MJ showed intermediate contents of total saponins. Based on the findings from **Figures 2A, B**, optimal concentrations of auxins and cytokinins along with the optimal concentration of squalene were used in combinations and results are presented in **Figure 2C**. It was revealed that the combination of cytokinins, auxins, and precursors together enhanced the total saponin content in cultures of *B. floribunda*. At the same combinations studied, TSC was found in the range of 20 to 36 mg/g DW wherein MS + 2.0 mg/l BAP + 1.5 μM SQ revealed the highest content of saponins (35.95 ± 0.022 mg DE/g DW) (**Figure 2C**). Furthermore, 10-week-old hardened plants of *B. floribunda* had comparatively lower TSC (27.71 ± 0.045 mg DE/g DW). From the findings, it was clearly shown that elicitor and precursor feeding increased the TSC amount in the *in vitro* grown biomass of *B. floribunda*. These treatments showed the reliable amount of TSC from the *in vitro* grown biomass of *B. floribunda* and also indicated that it could be a good source of saponins (**Figures 2A–C**).

### Estimation of triterpenoid saponin glycosides using HPLC

**Figure 3:**
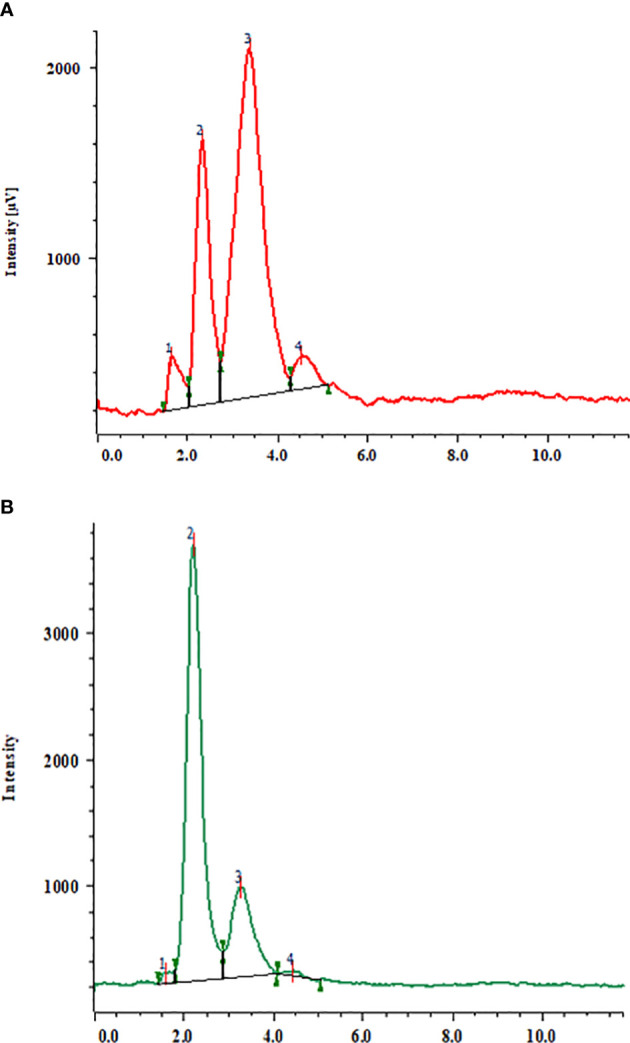
HPLC chromatogram of a mixture of triterpenoid saponin glycosides, **(A)** (1) bacoside A3; (2) bacopaside II; 3) bacopaside X; 4) bacopasaponin C and **(B)**, triterpenoid saponin glycosides from *in vitro* grown biomass (MS + 2.0 mg/l BAP + 1.5 μM SQ).

In the current study, an HPLC analysis of triterpenoid saponin glycosides (bacoside A3, bacoside II, bacopaside X, and bacosaponin C) from the *in vitro* grown biomass and hardened plants of *B. floribunda* was carried out. The combination of jujubogenin (bacoside A3 and bacopaside X) and pseudojujubogenin (bacopaside II and bacosaponin C) glycosides was considered as total triterpenoid saponin glycosides. MS media fortified with various concentrations of PGRs, precursor, and elicitors and combinations of their optimal concentrations were examined. Additionally, a total of four triterpenoid saponin glycoside compounds were separated successfully and results are presented in **Figure 3A**. Extracts of *in vitro* grown biomass raised on MS medium fortified with KIN denoted a significant amount of total triterpenoid saponin glycosides (2 to 12 mg/g DW), followed by BA, NAA, IBA, and IAA. MS + 2.0 mg/l KIN revealed the highest (11.98 ± 0.10 mg/g DW) content of saponins followed by MS + 2.0 mg/l BAP (11.74 ± 0.10 mg/g DW). Likewise in the case of auxins studied, MS + 1.5 mg/l NAA showed the highest saponin content (7.61 ± 0.10 mg/g DW). In contrast, the least content (2.84 ± 0.10 mg/g DW) was observed when cultures were grown on MS + 1.5 mg/l IAA (**Figure 2A**). Furthermore, among all the tested concentrations of precursor and elicitors, MS + 1.5 μM SQ was identified as the best source of total triterpenoid saponin glycosides (6.42 ± 0.10 mg/g DW) as compared with its remaining concentrations wherein the least content (2.36± 0.10 mg/g DW) was denoted from the lowest level (0.5 μM) of squalene (**Figure 2B**). In elicitors, MS + 60 mM KCl indicated the highest saponin content (6.23 ± 0.10 mg/g DW), whereas incorporation of 30 μM MJ to the medium denoted the lowest amount (1.34 ± 0.10 mg/g DW) **(** **Figure 2B** **)**. The triterpenoid saponin glycosides are said to be the active chemical constituents reported from the genus *Bacopa* and mainly categorized as pseudojujubogenin (bacoside II and bacosaponin C) and jujubogenin glycosides (bacoside A3 and bacopaside X). The effects of different PGRs on production of triterpenoid saponin glycosides (jujubogenin glycoside and pseudojujubogenin glycoside) from *in vitro* grown biomass of *Bacopa floribunda* were investigated, and results are depicted in **Table 5**. In the present investigation, different treatments of PGRs showed variable responses wherein total jujubogenin and pseudojujubogenin glycoside contents were found in the range of 0.62 to 2.20 and 1.91 to 9.99 mg/g DW, respectively. Moreover, the highest total jujubogenin glycoside content (2.20 ± 0.10 mg/g DW) was observed from the biomass derived from MS + 2.0 mg/l BAP. Similar to this, maximum bacopaside X (2.150 ± 0.069 mg/g DW) and bacoside A3 (0.081 ± 0.019 mg/g DW) were reported from the auxins containing media (MS + 2.0 mg/l BAP and MS + 1.0 mg/l BAP, respectively). Further, the highest total pseudojujubogenin glycosides (9.99 ± 0.10 mg/g DW) were reported from the cultures derived from MS + 2.0 mg/l KIN. At the same time, maximum bacopaside II (9.93 ± 0.27 mg/g DW) and bacosaponin C (0.142 ± 0.090 mg/g DW) were reported from MS medium enriched with 2 mg/l KIN and 1.5 mg/l BAP, respectively. The addition of 1.5 mg/l IBA in the nutrient medium produced the highest amount of total jujubogenin glycoside content (1.429 ± 0.010 mg/g DW) wherein a maximum content of bacopaside X (1.425 ± 0.018 mg/g DW) was noted. In a similar way, MS + 0.5 mg/l IAA revealed the best results in terms of bacoside A3 content (0.206 ± 0.001 mg/g DW). The highest total pseudojujubogenin glycoside content (6.20 ± 0.10 mg/g DW) was obtained from the biomass derived from MS + 1.5 mg/l NAA (**Table 5** **).** In the current findings, we noted that bacopaside X and bacopaside II were the main ingredients found responsible for enhancing total jujubogenin and pseudojujubogenin glycosides in *B. floribunda*. Like PGRs, the influence of precursor and elicitors on production of bioactive compounds was also studied (**Table 6**). In the case of precursor studies, MS + 1.5 μM SQ denoted the highest total jujubogenin (0.936 ± 0.10 mg/g DW) and total pseudojujubogenin glycoside (5.49 ± 0.10 mg/g DW) contents. Among the studied elicitors, all the concentrations denoted a reliable amount of total jujubogenin and pseudojujubogenin glycosides, where MS + 80 μM MJ displayed the highest total jujubogenin glycoside content (0.878 ± 0.10 mg/g DW). Total pseudojujubogenin glycoside content was comparatively found to be higher (5.419 ± 0.10 mg/g DW) when 60 mM KCl was added to the medium. In the same way, MS + 80 mM KCl exhibited the highest bacoside A3 (0.020 ± 0.017 mg/g DW) content; however, bacopaside X content was maximum when the shoots were treated with 80 μM MJ (0.873 ± 0.049 mg/g DW). Similarly, the highest content of bacopaside II (4.242 ± 0.145 mg/g DW) was reported from the culture derived from the medium supplemented with 30 mM MgSO_4_ (**Table 6** **).**

**Table 5:** Effects of different PGRs on total triterpenoid saponin glycosides (jujubogenin glycoside and pseudojujubogenin glycoside) from *in vitro* grown biomass of *Bacopa floribunda*.

PGRs (mg/l)		Total triterpenoid saponin glycosides (mg/g DW)						
		Jujubogenin glycoside		Total Jujubogenin glycoside	Pseudojujubogenin glycoside		Total Pseudojujubogenin glycosides	Total jujubogenin and pseudojujubogenin glycosides
		Bacoside A3	Bacopaside X		Bacopaside II	Bacosaponin C		
**BAP**	0.5	0.032 ± 0.01^c^	0.588 ± 0.12^e^	0.62 ± 0.10^e^	2.320 ± 0.02^l^	0.034 ± 0.002^b^	2.35 ± 0.10°	2.97 ± 0.10^f^
	1.0	0.081 ± 0.019^b^	0.663 ± 0.120^de^	0.74 ± 0.10^e^	1.862 ± 0.098^m^	0.048 ± 0.001^b^	1.91 ± 0.10^q^	2.65 ± 0.10^f^
	1.5	0.041 ± 0.019^c^	1.913 ± 0.059^a^	1.95 ± 0.10^a^	8.814 ± 0.27^c^	0.142 ± 0.090^a^	8.96 ± 0.10^b^	10.91 ± 0.10^b^
	2.0	0.050 ± 0.002^b^	2.150 ± 0.069^a^	2.20 ± 0.10^a^	9.504 ± 0.270^b^	0.038 ± 0.014^b^	9.54 ± 0.10^b^	11.74 ± 0.10^a^
**KIN**	0.5	0.023 ± 0.01^d^	1.014 ± 0.042^c^	1.04 ± 0.10^c^	6.169 ± 0.155^d^	0.060 ± 0.024^b^	6.23 ± 0.10^c^	7.27 ± 0.10^c^
	1.0	0.025 ± 0.001^d^	0.909 ± 0.140^cd^	0.93 ± 0.10^e^	3.337 ± 0.085^i^	0.061 ± 0.028^b^	3.40 ± 0.10^k^	4.33 ± 0.10^d^
	1.5	0.026 ± 0.001^d^	1.309 ± 0.127^b^	1.33 ± 0.010^c^	5.498 ± 0.003^e^	0.065 ± 0.040^b^	5.56 ± 0.10^d^	6.90 ± 0.10^c^
	2.0	0.013 ± 0.001^d^	1.972 ± 0.101^a^	1.99 ± 0.10^a^	9.934 ± 0.276^a^	0.060 ± 0.038^b^	9.99 ± 0.10^a^	11.98 ± 0.10^a^
**IAA**	0.5	0.206 ± 0.001^a^	0.542 ± 0.380^e^	0.749 ± 0.10^d^	3.825 ± 0.115^h^	0.020 ± 0.009^b^	3.84 ± 0.10^i^	4.59 ± 0.10^d^
	1.0	0.035 ± 0.034^c^	0.761 ± 0.319^cde^	0.796 ± 0.10^d^	5.738 ± 0.192^e^	0.023 ± 0.001^b^	5.76 ± 0.10^c^	6.55 ± 0.10^c^
	1.5	0.059 ± 0.054^b^	0.574 ± 0.152^e^	0.633 ± 0.10^d^	2.178 ± 0.125^l^	0.029 ± 0.004^b^	2.20 ± 0.10^p^	2.84 ± 0.10^f^
	2.0	0.044 ± 0.039^c^	0.786 ± 0.097^cde^	0.830 ± 0.10^d^	2.870 ± 0.112^k^	0.059 ± 0.001^b^	2.92 ± 0.10^n^	3.75 ± 0.10^e^
**IBA**	0.5	0.018 ± 0.015^d^	0.971 ± 0.083^c^	0.989 ± 0.10^c^	3.039 ± 0.079^jk^	0.055 ± 0.018^b^	3.09 ± 0.10^m^	4.08 ± 0.10^c^
	1.0	0.002 ± 0.001^e^	0.972 ± 0.084^c^	0.974 ± 0.010^c^	4.111 ± 0.139^g^	0.055 ± 0.019^b^	4.16 ± 0.10^h^	5.14 ± 0.10^d^
	1.5	0.004 ± 0.002^e^	1.425 ± 0.018^b^	1.429 ± 0.010^b^	5.290 ± 0.094^e^	0.053 ± 0.015^b^	5.34 ± 0.10^e^	6.77 ± 0.10^c^
	2.0	0.005 ± 0.001^e^	1.263 ± 0.237^b^	1.268 ± 0.10^b^	3.753 ± 0.018^h^	0.060 ± 0.023^b^	3.81 ± 0.10	5.08 ± 0.10^d^
**NAA**	0.5	0.003 ± 0.002^e^	0.894 ± 0.045^cd^	0.897 ± 0.10^c^	4.578 ± 0.025^f^	0.046 ± 0.007^b^	4.62 ± 0.10^g^	5.52 ± 0.10^d^
	1.0	0.004 ± 0.001^e^	0.819 ± 0.025^cde^	0.823 ± 0.10^c^	3.162 ± 0.066^ij^	0.044 ± 0.014^b^	3.20 ± 0.10^l^	4.02 ± 0.10^c^
	1.5	0.004 ± 0.001^e^	1.404 ± 0.044^b^	1.407 ± 0.10^b^	6.165 ± 0.056^d^	0.043 ± 0.001^b^	6.20 ± 0.10^c^	7.61 ± 0.10^c^
	2.0	0.004 ± 0.002^e^	1.298 ± 0.024^b^	1.302 ± 0.10^b^	4.667 ± 0.328^f^	0.046 ± 0.005^b^	4.71 ± 0.10^f^	6.01 ± 0.10^c^

Values represent the averages and standard errors of three independent measurements. According to Duncan’s multiple-range test, mean values in the same column with various alphabets exhibited statistically significant differences.

**Table 6:** Effects of precursor and elicitors on total triterpenoid saponin glycosides (jujubogenin glycoside and pseudojujubogenin glycoside) from *in vitro* grown biomass of *Bacopa floribunda*.

Precursor/elicitors		Total triterpenoid saponin glycosides (mg/g DW)						
		Jujubogenin glycoside		Total jujubogenin glycoside	Pseudojujubogenin glycosides		Total pseudojujubogenin glycoside	Total amount of jujubogenin and pseudojujubogenin glycosides
		Bacoside A3	Bacopaside X		Bacopaside II	Bacosaponin C		
**SQ** **(μM)**	0.5	0.011 ± 0.007^b^	0.476 ± 0.177^ijkl^	0.487 ± 0.10e	1.835 ± 0.412^jkl^	0.044 ± 0.029^b^	1.879 ± 0.10^d^	2.366 ± 0.10^d^
	1.0	0.014 ± 0.006^b^	0.519 ± 0.082^hijk^	0.533 ± 0.10^d^	1.902 ± 0.086^hij^	0.037 ± 0.022^c^	1.939 ± 0.10^d^	2.472 ± 0.10^d^
	1.5	0.005 ± 0.003^b^	0.930 ± 0.088^a^	0.936 ± 0.10^a^	5.463 ± 0.211^a^	0.031 ± 0.003^c^	5.493 ± 0.10^a^	6.429 ± 0.10^a^
	2.0	0.045 ± 0.041^a^	0.833 ± 0.028^ab^	0.878 ± 0.10^b^	4.673 ± 0.119^bc^	0.020 ± 0.001^c^	4.693 ± 0.10^b^	5.571 ± 0.10^b^
**MJ** **(μM)**	30	0.011 ± 0.006^b^	0.418 ± 0.046^kl^	0.429 ± 0.10^e^	0.872 ± 0.045^m^	0.043 ± 0.016^b^	0.915 ± 0.10^f^	1.344 ± 0.10^f^
	60	0.006 ± 0.004^b^	0.581 ± 0.098^efgh^	0.587 ± 0.10^d^	2.125 ± 0.078^ghi^	0.052 ± 0.024^b^	2.177 ± 0.10^d^	2.764 ± 0.10^d^
	80	0.005 ± 0.001^b^	0.873 ± 0.049^ab^	0.878 ± 0.10^a^	3.880 ± 0.020^d^	0.079 ± 0.042^a^	3.959 ± 0.10^c^	4.837 ± 0.10^c^
	100	0.008 ± 0.001^b^	0.497 ± 0.007^kl^	0.505 ± 0.10^d^	1.551 ± 0.037^l^	0.067 ± 0.022^a^	1.618 ± 0.10^e^	2.123 ± 0.10^d^
**SA** **(μM)**	30	0.003 ± 0.002^b^	0.611 ± 0.038^efg^	0.614 ± 0.10^c^	3.585 ± 0.027^e^	0.027 ± 0.001^c^	3.612 ± 0.10^c^	4.226 ± 0.10^c^
	60	0.005 ± 0.001^b^	0.518 ± 0.017^hijk^	0.523 ± 0.10^d^	2.810 ± 0.058^f^	0.016 ± 0.004^d^	2.826 ± 0.10^d^	3.349 ± 0.10^d^
	80	0.009 ± 0.003^b^	0.644 ± 0.031^efg^	0.653 ± 0.10^c^	3.605 ± 0.024^e^	0.014 ± 0.008^d^	3.619 ± 0.10^d^	4.273 ± 0.10^c^
	100	0.011 ± 0.002^b^	0.397 ± 0.021^l^	0.408 ± 0.10^e^	1.485 ± 0.039^l^	0.023 ± 0.005^c^	1.507 ± 0.10^e^	1.915 ± 0.10^e^
**KCl** **(mM)**	30	0.005 ± 0.001^b^	0.683 ± 0.076^def^	0.688 ± 0.10^c^	3.498 ± 0.098^e^	0.022 ± 0.006^c^	3.521 ± 0.10^c^	4.208 ± 0.10^c^
	60	0.004 ± 0.001^b^	0.816 ± 0.061^bc^	0.820 ± 0.10^a^	5.406 ± 0.041^a^	0.013 ± 0.001^d^	5.419 ± 0.10^a^	6.239 ± 0.10^a^
	80	0.020 ± 0.017^b^	0.738 ± 0.028^cde^	0.759 ± 0.10^b^	4.212 ± 0.125^c^	0.019 ± 0.005^d^	4.231 ± 0.10^b^	4.989 ± 0.10^c^
	100	0.005 ± 0.002^b^	0.552 ± 0.001^ghi^	0.557 ± 0.10^d^	2.756 ± 0.103^f^	0.018 ± 0.004^d^	2.774 ± 0.10^e^	3.331 ± 0.10^d^
**MgSO_4_** **(mM)**	30	0.009 ± 0.001^b^	0.773 ± 0.110^bcd^	0.782 ± 0.10^b^	4.242 ± 0.145^c^	0.058 ± 0.044^b^	4.301 ± 0.10^b^	5.082 ± 0.10^a^
	60	0.006 ± 0.001^b^	0.432 ± 0.009^jkl^	0.438 ± 0.10^e^	1.957 ± 0.167^hij^	0.019 ± 0.003^d^	1.976 ± 0.10^e^	2.414 ± 0.10^d^
	80	0.007 ± 0.001^b^	0.575 ± 0.03^defgh^	0.582 ± 0.10^d^	3.387 ± 0.550^e^	0.029 ± 0.010^c^	3.416 ± 0.10^c^	3.998 ± 0.10^c^
	100	0.011 ± 0.003^b^	0.409 ± 0.01^ghijkl^	0.419 ± 0.10^e^	1.534 ± 0.086^kl^	0.017 ± 0.005^d^	1.551 ± 0.10^e^	1.970 ± 0.10^e^
**Hardened plantlet**		0.001 ± 0.001^c^	1.48 ± 0.001^a^	1.485 ± 0.01^a^	1.59 ± 0.001^kl^	0.67 ± 0.001^a^	2.27 ± 0.001^e^	3.754 ± 0.001^c^

Values represent the averages and standard errors of three independent measurements. According to Duncan’s multiple-range test, mean values in the same column with various alphabets exhibited statistically significant differences.

Furthermore, optimal concentrations of auxins and cytokinins along with squalene were used in combinations to boost the total amount of triterpenoid saponin glycosides. Among all the optimum tested concentrations, MS + 2.0 mg/l BAP + 1.5 μM SQ was found to be the best with the highest content of total triterpenoid saponin glycosides (46.04 ± 0.10 mg/g DW) (**Figure 3B** **)**. Similarly, other remaining optimal combinations revealed total saponins in the range of 24 to 46 mg/g DW, wherein the total jujubogenin glycoside content was 5.69 ± 0.10 mg/g DW from MS + 2.0 mg/l BAP + 2.0 mg/l KIN. Similarly, MS + 2.0 mg/l BAP + 1.5 μM SQ was identified as the best treatment and offered the highest total pseudojujubogenin glycosides (43.80 ± 0.10 mg/g DW). Among all the triterpenoid saponin glycosides, the highest bacoside A3 content (1.23 ± 0.064 mg/g DW) was obtained from the biomass grown on MS + 0.5 mg/l IAA + 0.5 mg/l IBA + 1.0 NAA mg/l + 1.5 μM SQ (**Table 7**, **Figure 2C**). Among the triterpene saponins studied, maximum bacopaside X (4.95 ± 0.58 mg/g DW) was found when the nutrient medium was enriched with BAP and KIN (MS + 2.0 mg/l BAP + 2.0 mg/l KIN). Similarly, higher levels of bacopaside II (34.96 ± 2.7 mg/g DW) and bacosaponin C (0.29 ± 0.148 mg/g DW) were reported from the *in vitro* biomass grown on combinations of auxins and cytokinins (MS + 1.0 mg/l NAA + 2.0 mg/l KIN and MS + 2.0 mg/l BAP + 2.0 mg/l KIN, respectively). In the same way, 10-week-old hardened plants of *B. floribunda* also highlighted the appreciable amount of saponins (3.75 ± 0.001 mg/g DW) with higher levels of bacopaside X and bacopaside II (1.48 ± 0.001 and 1.59 ± 0.001 mg/g DW, respectively). These results revealed that the combinations of PGRs and the precursor (SQ) at its optimum concentrations exhibited the highest amount of triterpenoid saponin glycosides. According to the data, it was established that *B. floribunda* could be a reliable source of triterpenoid saponin glycosides and may serve as an alternative and potential candidate to the commonly used *B. monnieri* (**Tables 5**–**7** **;** **Figures 2A–C**).

**Table 7:** Effects of optimal concentrations of PGRs and precursor combinations on total triterpenoid saponin glycosides (jujubogenin glycoside and pseudojujubogenin glycoside) from *in vitro* grown biomass of *Bacopa floribunda*.

PGRs and precursor (mg/l–μM)	Total triterpenoid saponin glycosides (mg/g DW)						
	Jujubogenin glycoside		Total jujubogenin glycoside	Pseudojujubogenin glycoside		Total Pseudojujubogenin glycosides	Total amount of jujubogenin and pseudojujubogenin glycosides
	Bacoside A3	Bacopaside X		Bacopaside II	Bacosaponin C		
OCC^*^	0.74± 0.066^b^	4.95 ± 0.587^a^	5.69 ± 0.10^a^	31.57 ± 0.966^c^	0.29 ± 0.148^a^	31.86 ± 0.10^c^	37.55 ± 0.10^c^
OCA^*^	0.98 ± 0.120^a^	2.84 ± 0.103^b^	3.81 ± 0.10^d^	31.11 ± 1.19^c^	0.22 ± 0.117^a^	31.33 ± 0.10^c^	35.15 ± 0.10^d^
OCC^*^P**^°^**	1.16± 0.058^a^	2.93 ± 0.754^b^	4.08 ± 0.10^b^	20.17 ± 1.2^e^	0.15 ± 0.117^a^	20.31 ± 0.10^e^	24.39 ± 0.10^h^
OCA^*^P**^°^**	1.23 ± 0.064^a^	2.51 ± 0.419^bc^	3.75 ± 0.10^e^	25.77 ± 2.2^d^	0.27 ± 0.07^a^	26.04 ± 0.10^d^	29.79 ± 0.10^f^
OCC^*^P1^°^	1.01 ± 0.195^a^	1.23 ± 0.570^d^	2.24 ± 0.10^h^	43.62 ± 0.657^a^	0.19 ± 0.195^a^	43.80 ± 0.10^a^	46.04 ± 0.10^a^
OCC^*^P2**^°^**	1.09 ± 0.195^a^	2.76 ± 0.43^bc^	3.84 ± 0.10^c^	30.77 ± 2.00^c^	0.17 ± 0.053^a^	30.94 ± 0.10^c^	34.79 ± 0.10^e^
OCAC^*^P**^°^**	0.99 ± 0.157^a^	1.68 ± 1.08^cd^	2.67 ± 0.10^g^	24.64 ± 1.59^d^	0.13 ± 0.040^a^	24.78 ± 0.10^d^	27.45 ± 0.10^g^
OCAC^*^	1.11 ± 0.127^a^	2.16 ± 0.37^bc^	3.27 ± 0.10^f^	34.96 ± 2.7^b^	0.13 ± 0.040^a^	35.09 ± 0.10^b^	38.36 ± 0.10^b^

^*^(mg/l), **^°^** (μM), OCC^*^ - MS + 2.0 mg/l BAP + 2.0 mg/l KIN, OCA^*^ - MS + 0.5 mg/IAA + 0.5 mg/IBA + 1.0 mg/l NAA, OCC^*^P^°^ - MS + 2.0 mg/BAP + 2.0 mg/l KIN + 1.5 μM SQ, OCA^*^P^°^ - MS + 0.5 mg/l IAA + 0.5 mg/l IBA + 1.0 mg/l NAA + 1.5 μM SQ, OCC^*^P1^°^- MS + 2.0 BAP mg/l + 1.5 μM SQ, OCC^*^P2^°^- MS + 2.0 mg/l KIN + 1.5 μM SQ, OCAC^*^P^°^ - MS + 1.0 mg/l NAA + 2.0 mg/l KIN + 1.5 μM SQ, OCAC^*^ - MS + 1.0 mg/l NAA + 2.0 mg/l KIN.

Values represent the averages and standard errors of three independent measurements. According to Duncan’s multiple-range test, mean values in the same column with various alphabets exhibited statistically significant differences.

### Estimation of stigmasterol content

**Figure 4:**
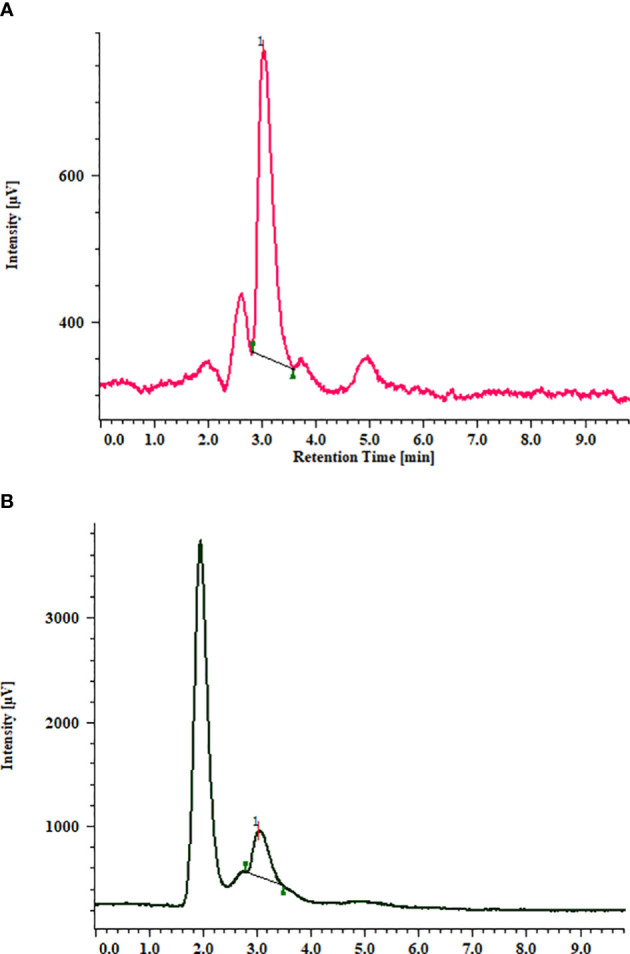
HPLC chromatogram of standard stigmasterol **(A)**. Stigmasterol from *in vitro* grown biomass of MS + 2.0 mg/l BAP **(B)**.

Similar to total triterpenoid saponin glycosides, stigmasterol content from the *in vitro* grown biomass of *Bacopa floribunda* was studied using HPLC (**Figures 2** and **4**). Separation of compound was carried out using acetonitrile and water (70:30 v/v), and the compound was detected at 3.01 ± 0.1 min **(** **Figure 4A** **)**. In the cytokinin study, biomass obtained from MS nutrient medium containing 2.0 mg/l BAP had the highest stigmasterol content (7.69 ± 0.102 mg/g DW), whereas the least was found in the case of MS + 0.5 mg/l BAP (1.39 ± 0.31 mg/g DW). Similar to cytokinins, addition of 1.5 mg/l IBA to the MS medium denoted the maximum stigmasterol content (2.31 ± 0.66 mg/g DW) and the lowest in MS + 1.0 mg/l NAA (0.98 ± 0.17 mg/g DW) (**Figure 2A**). In precursor studies, incorporation of 1.5 μM SQ offered the best response (1.14 ± 0.13 mg/g DW) followed by MS + 1.0 μM SQ (1.27 ± 0.043 mg/g DW), 2.0 μM SQ (0.91 ± 0.069 mg/g DW), and 0.5 μM SQ (0.82 ± 0.034 mg/g DW), respectively (**Figure 2B**). Among all the elicitors studied, the content of stigmasterol was noted in the range of 0.72 to 1.51 mg/g DW. Utilization of 80 μM MJ in the medium revealed the highest stigmasterol content (1.51 ± 0.10 mg/g DW), whereas the minimum was found when biomass was grown on MS + 100 mM SA. An intermediate content of stigmasterol (0.72 to 1.43 mg/g DW) was observed when the cultures were raised using remaining elicitors such as SA, KCl, and MgSO_4_. Further, combinations of optimum concentrations were studied and all the tested treatments showed comparatively less amount (1.6 to 2.6 mg/g DW) of stigmasterol (**Figure 2C**). A similar trend was noted in the case of 10-week-old hardened plants (3.71 ± 0.106 mg/g DW). From the above results, it can be concluded that MS along with 2.0 mg/l BAP showed an elevated level of stigmasterol as compared with the other treatments (**Figures 2A–C** and **4B** **)**. These findings indicated that all the PGRs, precursor, and elicitors and the combinations of their optimal concentrations displayed a significant amount of stigmasterol and also highlighted that *B. floribunda* could be a reliable source of phytosterol (**Figure 2**).

### Chemometric analysis

**Figure 5:**
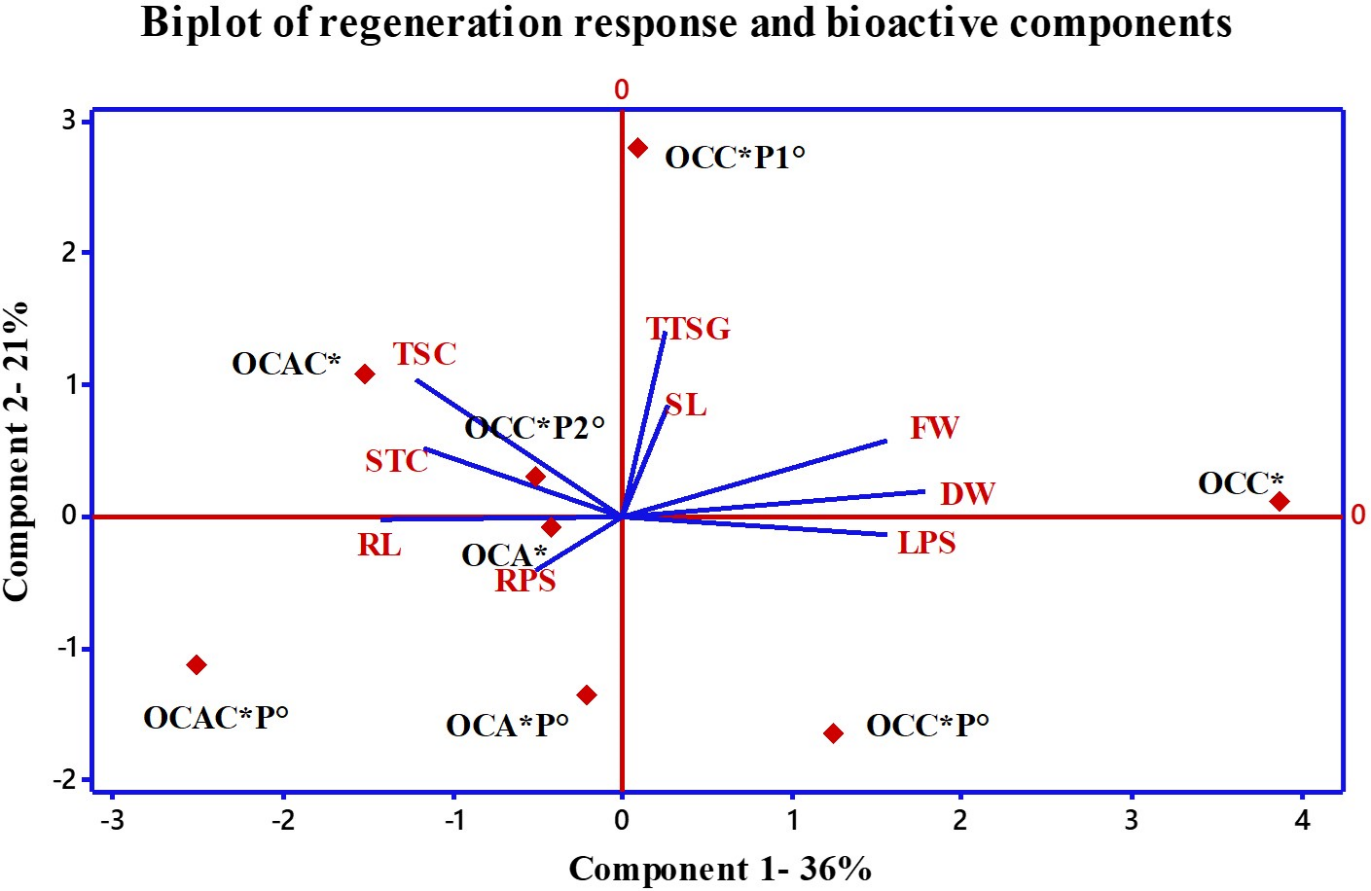
Principal component analysis (scores and loading plots, biplot) of optimal combinations of PGRs with precursors on growth and production of bioactive compounds from *B*. *floribunda.* *mg/l; °μM. SL, shoot length; LPS, leaves per shoot; RL, root length; RPS, roots per shoot; FW, fresh weight; DW, dry weight; TSC, total saponin content; STC, stigmasterol content; TTSG, total triterpenoid saponin glycosides; OCC, optimal combination of cytokinins; OCCP1, optimal combination of cytokinin with precursor; OCCP2, optimal combination of cytokinin with precursor; OCA, optimal combination of auxins; OCAP, optimal combination of auxin with precursor; OCACP, optimal combination of auxin, cytokinin, and precursor; OCAC, optimum combination of auxins with cytokinin; OCCP, optimum combination of cytokinin with precursor.

PCA was performed to understand the relationship between the studied morphological responses along with detected bioactive compounds (total saponins, stigmasterol, and triterpenoid saponin glycosides). In the analysis, responses from various optimal concentrations of PGRs and precursors and their combinations were used (**Figure 5**). Multivariate analysis showed that components 1 and 2 highlighted 36 and 21% variability, respectively. **Figure 5** also shows that the positive planes of both components were occupied by TBC (0.07), FW (0.42), DW (0.49), and SL (0.07). Optimal combination of cytokinins (OCC) and optimal combination of cytokinin and precursor (OCCP1) enjoyed the positive plane of both the components. Only parameter LPS having a loading value of 0.42 occupied the positive plane of PC1 and the negative plane of PC2. Similarly, component 2 was mainly dominated by TTSG and STC with 0.48 and 0.23 loading values, respectively. OCCP2 and OCAC enjoyed the positive plane of component 2. From **Figure 5**, it was clearly shown that OCA along with RL and RPS occupied the negative planes of PC1 and PC2. Treatments like OCACP, OCAP, and OCCP were found less effective for the regeneration responses. However, results also highlighted that treatments like OCAC, OCCP2, and OCA contributed toward the higher contents of total saponins, total triterpenoid saponin glycosides, and stigmasterol (**Figure 5**).

## Discussion

*Bacopa* species are mainly found in wetland areas of tropical and subtropical countries, and its growth under natural conditions is limited. In order to increase mass multiplication of *B. floribunda* and secondary metabolites including triterpenoid saponin glycosides and sterols, it is necessary to develop an efficient *in vitro* micropropagation protocol. In order to accomplish this goal, we focused on micropropagation and augmented production of stigmasterol and triterpenoid saponin glycosides from the unexplored plant species known as *B. floribunda.* Owing to their enormous potential in medicinal purposes, most of *B. monnieri* have been collected extensively from their native habitat, resulting in the species’ extinction and threat. Search of alternate herbs and their mass multiplication is also needed to reduce the overexploitation of *B. monnieri*. To achieve this goal, we aimed at micropropagation and elicited production of triterpenoid saponin glycosides as well as stigmasterol in *B. floribunda*. Seeds of *B. monnieri* had poor germination, low viability, and frequent two-leaf seedling deaths, but it can be propagated efficiently using stem cuttings (Parale and Nikam, 2009; Parale et al., 2010). In the study, fresh nodal segments were used as explants and cultures were established using MS medium supplemented with varied concentrations of auxins and cytokinins. The present investigation revealed that MS supplemented with PGRs (BAP, KIN, IAA, IBA, and NAA) alone and in combination revealed the formation of multiple shoots and roots in *B. floribunda*. Our study revealed optimal regeneration responses when the MS medium was supplemented with cytokinins such as KIN and BAP. These findings are in accordance with earlier reports where researchers suggested that the highest concentrations of cytokinins promoted the regeneration of *B. monnieri* through axillary nodes and internodes (Banerjee and Shrivastava, 2008). In addition, several researchers also reported that BAP was found to be the most effective in cytokinin, which played an important role in induction of multiple shoots in *B. monnieri* (Tiwari et al., 2001; Ceasar et al., 2010). According to Banerjee and Shrivastava (2008), internode explants of *B. monnieri* revealed a significantly higher number of shoots when inoculated into MS media supplemented with 1.0 mg/l BAP and 0.5 mg/l KIN. Our findings are in good agreement with Parale and Nikam (2009) who observed that BA and KIN at concentrations of 1 to 10 μM improved the shoot regeneration in *B. monnieri*. In all the auxin treatments, incorporation of 1.0 mg/l NAA in nutrient medium showed optimum root regeneration (Banerjee and Shrivastava, 2008). Researchers reported that roots of *B. monnieri* showed optimum growth when MS medium was supplemented with 1.5 mg/l NAA. It is a fact that PGRs such as cytokinins (BAP and KIN) stimulate the initiation and further elongation of shoots whereas auxins (IAA, IBA, and NAA) promoted the root initiation, growth, and development. In addition, the composition of nutrient media used in *in vitro* propagation also affected the plant growth and development (Komakech et al., 2020). Plant cell culture growth is mainly affected by nutrient concentration, stress variables, photoperiod, light quality, genotype, and concentration as well as combinations of plant growth regulators (Chavan et al., 2010).

The present study reported that among the precursor and elicitors used, utilization of 1.5 μM SQ in MS medium showed optimum regeneration responses. In accordance with our studies, Baek et al. (2019) also reported that squalene at different concentrations (up to 10 mM) increased the growth index of hairy roots of *Centella asiatica*. Likewise, elicitors at various concentrations altered the growth performance and the best response was observed when 80 μM MJ was added to the nutrient medium. Furthermore, cultures of *B. floribunda* grown on MS nutrient medium with methyl jasmonate, salicylic acid, potassium chloride, and magnesium chloride also flourished well. In the same way, Sharma et al. (2015) reported that abiotic elicitors (jasmonic acid, copper sulfate, and salicylic acid) stimulated the biomass production in *in vitro* shoot cultures of *B. monnieri*. Moreover, Largia et al. (2015) studied the influence of elicitors (methyl jasmonate, salicylic acid) on growth, biomass, and morphology of *B. monnieri*. Patil et al. (2013) also found that elicitation feeding improved the *in vitro* growth of *Digitalis purpurea*. Elicitors are compounds well known to increase the metabolic activities of phytochemicals and further improve plant performance. The plant physiology and biochemistry of the tissues are also affected by the type and/or concentration of the elicitor. Responses of the species also depend on the elicitor and its concentration added in the medium. In accordance with our findings, in plant cell cultures, the use of elicitors has been found to be beneficial (Guru et al., 2022). Additionally, a number of precursors and elicitors have been used earlier to induce morphological and physiological alterations in *in vitro* cultures (Patil et al., 2013; Watcharatanon et al., 2019). Optimal concentrations of PGRs, precursor, and elicitors alone and in combination were used to study the growth responses in *B. floribunda*. The goal of this experiment was to increase the production of *in vitro* biomass of *B. floribunda*. From the present investigations, we found that optimum cytokinin concentrations, i.e., MS + 2.0 mg/l KIN + 2.0 mg/l BAP, demonstrated the highest growth responses (average shoot length, number of leaves per shoot, FW, and DW). Martins et al. (2022) found that the combination of two cytokinins can be more advantageous than using only one, which is consistent with our findings. In our studies, also the combination of auxins (MS + 0.5 IAA mg/l + 0.5 IBA mg/l + 1.0 mg/l NAA) promoted the root initiation, elongation, and number of roots per shoot. According to earlier reports, rooting of the *in vitro* produced shoots of *B. monnieri* was higher when auxin-rich MS medium was used (Banerjee and Shrivastava, 2008; Sudheer et al., 2022). Selection of PGRs plays a crucial role in *in vitro* propagation of the plant. Cytokinins and auxins were commonly used in culture media to influence physiological reactions that led to promotion of the growth of shoots, roots, and whole plant. A number of researchers have investigated the impact of auxins (NAA, IAA) and cytokinins (BAP, KIN) on *in vitro* growth and secondary metabolites from the plant (Amoo and Staden, 2012; Martins et al., 2022). The highest concentrations of cytokinins offered the best outcomes, which may be due to the ability of BAP to promote cell proliferation and lateral bud development. It also plays a key role in altering the axillary bud dormancy. BAP exhibits a more sustained stimulation of cell division due to its stable nature and resistance to oxidation characteristics. In addition, it cannot easily broken down by plants (Komakech et al., 2020; Okello et al., 2021; Martins et al., 2022).

Well-developed plantlets were successfully acclimatized to the field conditions with a good survival rate of 95%, which was in agreement with earlier reports (Tiwari et al., 2001; Sharma et al., 2016; ). These plantlets were morphologically similar to the field-grown plants. Acclimatized plants may have aberrant anatomy, physiology, or morphology, which takes longer to adapt and lowers growth. From various tested concentrations of PGRs, precursor, and elicitors, a reliable amount of total saponin content was noted. In cytokinins, MS + 1.0 mg/l BAP showed the highest TSC (31.79 ± 0.011 mg DE/g DW), whereas, among the tested auxins, incorporation of IBA in the medium MS + 0.5 mg/l IBA proved to be the best (32.0 ± 0.001 mg DE/g DW). In our studies, addition of precursor SQ (1.5 μM) revealed the highest TSC. Likewise, among all the elicitors tested, incorporation of KCl (80 mM) to the nutrient medium offered the highest content of total saponins. Additionally, combination of cytokinins and squalene (MS + 2.0 mg/l BAP + 1.5 μM SQ) demonstrated the best results as compared with other treatments. Our findings are in good agreement with Phrompittayarat et al. (2011). Triterpenoid saponin glycosides of *B. monnieri* were earlier reported from the aerial parts as well as shoots. The saponin content may vary depending on several variables, including the plant components, environment, season, and harvesting time. Watcharatanon et al. (2019) reported improvement in total triterpenoid saponin glycosides when *in vitro* cultures of *B. monnieri* were fed with the precursors and exposed to light-emitting diode (LED). The study suggested that saponin content may be enhanced as a result of the feeding of precursors and LED light exposure to *in vitro* cultures of *B. monnieri*.

HPLC analysis confirmed the presence of four triterpenoid saponin glycosides (bacoside A3, bacopaside X, bacopaside II, and bacosaponin C) from the *in vitro* grown biomass treated with various PGRs, precursor, and elicitors as well as acclimatized plantlets. On the other hand, several researchers identified and quantified bacoside saponins from *B. monnieri* and also reported potent biological activities like anti-inflammatory, antioxidant, anti-hepatotoxic, and analgesic and superoxide inhibition (Bhandari et al., 2006; Sudheer et al., 2022). It is a classified and well-known drug used in the improvement of memory or mental power (Ganzera et al., 2004; Deepak et al., 2005; Bhandari et al., 2006; Naik et al., 2012; Saini et al., 2012; Bansal et al., 2014; Christopher et al., 2017; Bhandari et al., 2020).

Bacosides are the mixture of four saponin glycosides, *viz*., bacoside A3, bacopaside II, bacosaponin C, and bacopaside X, generally referred to as bacoside A (Nuengchamnong et al., 2016). Additionally, LC-ESI-QTOF-MS-based screening of the mixture of the authenticated saponin (bacoside A) also revealed that each of the individual compounds was identified individually by its mass as bacoside A3, bacopaside II, bacopaside X, and bacosaponin C. Additionally, total ion chromatogram (TIC) of the authenticated compounds proved that the bacoside mixture includes bacoside A3, bacopaside II, bacopaside X, and bacosaponin C (Nuengchamnong et al., 2016).

In the present findings, HPLC analysis confirmed the presence of four triterpenoid saponin glycosides in the *in vitro* grown biomass and acclimatized plantlets of *B. floribunda*. MS + 2.0 mg/l KIN alone highlighted the highest triterpenoid saponin glycosides, in which bacoside A3 (0.013 ± 0.001 mg/g DW), bacopaside II (9.934 ± 0.276 mg/g DW), bacopaside X (1.972 ± 0.101 mg/g DW), and bacosaponin C (0.060 ± 0.038 mg/g DW) contents were recorded. Similar to cytokinins tested, MS + 1.5 mg/l NAA revealed the highest contents of bacoside A3 (0.004 ± 0.001 mg/g DW), bacopaside II (6.16 ± 0.05 mg/g DW), bacopaside X (1.40 ± 0.044 mg/g DW), and bacosaponin C (0.043 ± 0.001 mg/g DW). Our results clarified that the higher concentrations of cytokinins (2 mg/l) proved to be the best and offered the highest triterpenoid saponin glycoside content. Our results are in agreement with reported results of Watcharatanon et al. (2019) who reported the highest triterpenoid saponin glycosides from *in vitro* grown cultures of *B. monnieri* (17.35 ± 0.13 mg/g DW). Likewise, Muzynska et al. (2016) also reported that shoots grown on MS medium supplemented with 1.0 mg/l BAP and 0.2 mg/l NAA revealed the highest bacoside A3 (2.18 ± 0.15 mg/g DW), bacopaside II (19.19 ± 1.1 mg/g DW), bacopaside X (0.67 ± 0.05 mg/g DW), and bacosaponin C (4.75 ± 0.01 mg/g DW) contents. Kamonwannasit et al. (2008) reported that shoots produced from internodal explants using MS + 0.5 mg/l TDZ showed remarkable pseudojujubogenin glycoside content (30.62 ± 1.29 mg/g DW). Among the different concentrations of the precursor used, MS medium enriched with 1.5 μM SQ revealed the highest total bacoside content (6.4 ± 0.10 mg/g DW). In accordance with our studies, Baek et al. (2019) also reported the enhancement of targeted triterpenoids in *Centella asiatica* hairy root cultures fed with 2.5 mM SQ. Dasari et al. (2020) also revealed that productivity of bioactive compounds depends on the concentration of chemicals used in the nutrient medium and also the exposure time. In *in vitro* grown biomass of *B. floribunda*, elicitors such as MJ, SA, KCl, and MgSO_4_ were found to be the most effective for the production of triterpenoid saponin glycosides. In line with our findings, elicitors like methyl jasmonate and salicylic acid enhanced the bacoside production in shoot cultures of *B. monnieri* (Largia et al., 2015). Sharma et al. (2015) noted enhancement of triterpenoid saponins from *B. monnieri* by utilizing methyl jasmonate as the key elicitor. Researchers also studied the influence of different elicitors (jasmonic acid, copper sulfate, and salicylic acid) on stimulation of bacoside production in shoot cultures of *B. monnieri*. Biotic (chitosan) and abiotic (salicylic acid, methyl jasmonate) elicitors directly affected the metabolite(s) production in medicinal plants (Kaur et al., 2022). Among all various studied elicitors, MJ and SA have been well documented for the increased production of triterpenoid saponins in plant cell cultures (Largia et al., 2015). As compared with previous reported results, we found satisfactory results from *in vitro* grown biomass of *B. floribunda* treated with MJ and SA. Elicitation is a technique of inducing or increasing the production of secondary metabolites in plant tissue culture (Kaur et al., 2022). According to the studies, elicitors may serve as signaling molecules in proper quantities, and when perceived by a plasma membrane receptor, they trigger a complex signal transduction network that regulates gene expression and leads to the production of the targeted compound(s). The complexity of elicitor signal transduction, the diversity and specificity of connections between elicitor signals and plant cell receptors, and subsequent plant cell defense responses may play a role in the variation in yield of targeted compounds (Chavan et al., 2010). As most of the elicitors are easily available in the market or can be produced and given to cell cultures in the laboratory, they can be used to produce secondary metabolites on a large scale (Chavan et al., 2010). Based on the aforesaid findings, an optimal level of the precursor was combined with the optimal concentrations of PGRs to get the maximum amount of metabolite accumulation. Surprisingly, we discovered that the combination of SQ and PGRs used in the medium promoted the bacoside content (46.04 ± 0.10 mg/g DW). When cytokinin (BAP) and precursor (SQ) were used in combination, it significantly enhanced total *Bacopa* saponins. Additionally, among all tested concentrations of elicitors and precursors, squalene was discovered to be the best. As SQ was found in the production route of saponins, it considerably improved the triterpenoid saponin glycosides. The mevalonate pathway is a crucial metabolic pathway with end products such as 3,3-dimethylallyl diphosphate (DMAPP) and isopentenyl diphosphate (IPP) that play a key mediating role in the formation of various secondary metabolites in plants. In order to produce different glycosylated triterpenoids, several enzymes participate in the structural alteration of these intermediates by substitution, oxidation, and glycosylation. Farnesyl pyrophosphate (FPP) molecules are converted to squalene by the enzyme squalene synthase, which is a key regulator in the biosynthesis of sterols and triterpenoids (Jeena et al., 2017; Murthy, 2022). According to earlier reports, *B. monnieri* was found to be an excellent source of bacosides. The results of our investigations showed that *in vitro* grown biomass of *B. floribunda* may serve as an alternate source of bacosides.

Additionally, stigmasterol is a subclass of phytosterols and has been shown to be preventative against cancer, diabetes, and hepatic and cardiovascular disorders. These sterols can reduce cancer risk by 20%, if included into the diet (Shahzad et al., 2017). Phytosterols have attracted the attention of researchers due to its diverse medicinal properties. In the current studies, cytokinin alone (2.0 mg/l BAP) showed the highest stigmasterol (7.69 ± 0.102 mg/g DW) content as compared with other levels of cytokinins. Similarly, MS supplemented with IBA alone (1.5 mg/l) showed the highest stigmasterol content (2.31 ± 0.665 mg/g DW). In precursor and elicitor studies, incorporation of squalene (1.5 μM) and methyl jasmonate (80 μM) in the nutrient medium revealed the maximum stigmasterol content (1.41 ± 0.13 and 1.51 ± 0.10 mg/g DW, respectively). In optimal concentrations of PGRs, elicitors, precursor, and their combinations (MS + 1.0 mg/l NAA + 2.0 mg/l KIN) denoted the maximum (2.56 ± 0.03 mg/g DW) stigmasterol content. These findings are in good agreement with Ghosh et al. (2011). The authors reported stigmasterol from the aerial portions of *B. monnieri*. It has been demonstrated that phytosterols have the ability to decrease cholesterol and also possess anticancer properties (Ghosh et al., 2011). Numerous secondary metabolites including stigmasterol also demonstrated potential biological functions and play a significant role in the food and pharmaceutical industries. Different plant parts have medicinal potential due to the presence of secondary metabolites such as phenolics, flavonoids, alkaloids, terpenoids, and sterols (Otari et al., 2022; Patel et al., 2022; Ghane et al., 2018; Lekhak et al., 2021). Many bioactive substances appear to have a variety of health benefits; thus, the demand for them in the food and pharmaceutical industries is high (Bhattacharyya et al., 2022; Otari et al., 2022). *B. floribunda* was also identified as a potential candidate with an additional source of bioactive constituents including triterpenoid saponin glycosides and stigmasterol.

In the present investigation, the correlation among studied optimal mixed concentrations with their respective regeneration responses along with bioactive metabolites (total saponins, triterpenoid saponin glycosides, and stigmasterol) was studied using a data reduction tool, i.e., PCA. The optimum concentration of cytokinin (MS + 2.0 mg/l BAP + 2.0 mg/l KIN) was identified as the best treatment that showed the highest shoot length, higher number of leaves per shoot, FW, and DW. Similarly, optimum mixed combinations of cytokinin with the precursor (MS + 2.0 mg/l BAP + 1.5 μM SQ) contributed toward the triterpenoid saponin glycosides. Similarly, the combination of auxins (MS + 0.5 mg/IAA + 0.5 mg/IBA + 1.0 mg/l NAA) revealed the highest root length and number of roots per shoot. According to the above findings, it can be concluded that cytokinins (BAP and KIN) were responsible for shoot growth whereas root initiation and multiple roots per shoot were mainly controlled by the combined treatment of optimal auxins (NAA, IBA, and IAA). Significant amounts of bioactive compounds were found in the optimal mixed combinations of cytokinin and precursor (MS + 2.0 mg/l BAP + 1.5 μM SQ). Jauhari et al. (2019) reported a strong correlation between total bacosides and phytochemical content due to incorporation of elicitors in the nutrient medium. It was noted that precursors play a vital role in boosting the bioactive potential in plants. Previous reports stated that PCA is the most powerful statistical method for evaluating the relationship between a large number of variables and condensing the data into small elements (Otari et al., 2022; Patel and Ghane, 2021; Ghane et al., 2018).

A thorough analysis confirmed the remarkable amount of the desired metabolites (total saponins, triterpenoid saponin glycosides, and stigmasterol) in *in vitro* grown biomass of *B. floribunda*. The favorable outcomes also underlined the goal of improving the production of secondary metabolites in the *in vitro* cultures of *B. floribunda* by using precursor and elicitor feeding. The current research work provides an appropriate approach for mass multiplication and also validates the biosynthesis and accumulation of secondary metabolites in *in vitro* grown biomass of *B. floribunda*. It forms the first report on *in vitro* propagation and elicited production of total saponins, triterpenoid saponin glycosides, and stigmasterol contents from *B. floribunda.* It could be a reliable source of nootropic drugs as well as an important substitute to *B. monnieri*.

## Conclusion

From the studies, we conclude that MS medium fortified with 2.0 mg/l BAP + 2.0 mg/l KIN was found to be the best for shoot development. Similarly, MS + 0.5 mg/l IAA + 0.5 mg/l IBA + 1.0 mg/l NAA promoted the root initiation and number of roots per shoots. The *in vitro* regenerated *B. floribunda* plants showed a survival rate of up to 95% after acclimatization and was found to be identical to the mother plant. The presence of triterpenoid saponin glycosides and stigmasterol in *in vitro* grown biomass was confirmed by HPLC, wherein MS + 2.0 mg/l BAP + 1.5 μM SQ offered the highest content of total pseudojujubogenin glycosides. Similarly, biomass obtained from MS medium fortified with 2.0 mg/l BAP exhibited the highest content of stigmasterol. The species must be conserved in order to produce valuable pharmaceuticals commercially (such as bacosides and stigmasterol), and its sustainable utilization is required. It may serve as a potential candidate and alternate substitute to the Indian Pennywort. Further, studies on detailed phytochemical diversity, pharmacological activities, and elicited production of bioactives using biotechnological tools are needed.

## Data availability statement

The raw data supporting the conclusions of this article will be made available by the authors, without undue reservation.

## Author contributions

SGG designed the experiment. The experiment was carried out by SSO, SPD, and SBP performed all the experiments. SO drafted the manuscript and had it critically reviewed by SGG. SSO, SBP and SGG took part in the statistical analysis and assisted in refining the manuscript. All authors contributed to the article and approved the submitted version.
